# Suggesting a holistic framework for understanding healthcare services leadership competence – a critical interpretive synthesis

**DOI:** 10.1108/LHS-08-2023-0059

**Published:** 2024-04-15

**Authors:** Ingrid Marie Leikvoll Oskarsson, Erlend Vik

**Affiliations:** Department of Business Administration and Social Sciences, Molde University College, Molde, Norway

**Keywords:** Health care, Health leadership competencies, Health services sector, Leadership, Management

## Abstract

**Purpose:**

Healthcare providers are under pressure due to increasing and more complex demands for services. Increased pressure on budgets and human resources adds to an ever-growing problem set. Competent leaders are in demand to ensure effective and well-performing healthcare organisations that deliver balanced results and high-quality services. Researchers have made significant efforts to identify and define determining competencies for healthcare leadership. Broad terms such as competence are, however, inherently at risk of becoming too generic to add analytical value. The purpose of this study is to suggest a holistic framework for understanding healthcare leadership competence, that can be crucial for operationalising important healthcare leadership competencies for researchers, decision-makers as well as practitioners.

**Design/methodology/approach:**

In the present study, a critical interpretive synthesis (CIS) was conducted to analyse competency descriptions for healthcare leaders. The descriptions were retrieved from peer reviewed empirical studies published between 2010 and 2022 that aimed to identify healthcare services leadership competencies. Grounded theory was utilised to code the data and inductively develop new categories of healthcare leadership competencies. The categorisation was then analysed to suggest a holistic framework for healthcare leadership competence.

**Findings:**

Forty-one papers were included in the review. Coding and analysing the competence descriptions resulted in 12 healthcare leadership competence categories: (1) character, (2) interpersonal relations, (3) leadership, (4) professionalism, (5) soft HRM, (6) management, (7) organisational knowledge, (8) technology, (9) knowledge of the healthcare environment, (10) change and innovation, (11) knowledge transformation and (12) boundary spanning. Based on this result, a holistic framework for understanding and analysing healthcare services leadership competencies was suggested. This framework suggests that the 12 categories of healthcare leadership competencies include a range of knowledge, skills and abilities that can be understood across the dimension personal – and technical, and organisational internal and – external competencies.

**Research limitations/implications:**

This literature review was conducted with the results of searching only two electronic databases. Because of this, there is a chance that there exist empirical studies that could have added to the development of the competence categories or could have contradicted some of the descriptions used in this analysis that were assessed as quite harmonised. A CIS also opens for a broader search, including the grey literature, books, policy documents and so on, but this study was limited to peer-reviewed empirical studies. This limitation could also have affected the result, as complex phenomenon such as competence might have been disclosed in greater details in, for example, books.

**Practical implications:**

The holistic framework for healthcare leadership competences offers a common understanding of a “fuzzy” concept such as competence and can be used to identify specific competency needs in healthcare organisations, to develop strategic competency plans and educational programmes for healthcare leaders.

**Originality/value:**

This study reveals a lack of consensus regarding the use and understanding of the concept of competence, and that key competencies addressed in the included papers are described vastly different in terms of what knowledge, skills and abilities they entail. This challenges the operationalisation of healthcare services leadership competencies. The proposed framework for healthcare services leadership competencies offers a common understanding of work-related competencies and a possibility to analyse key leadership competencies based on a holistic framework.

## Introduction

There are numerous reasons to the increased attention towards leadership competencies over the past decades (Aitken and Von Treuer, 2014; Liang *et al.*, 2020a, 2020b; Liang *et al.*, 2020a, 2020b; Murphy *et al.*, 2016; Pihlainen *et al.*, 2016). Health services across the world have been subject to continuous reform, reorganisation and restructuring since the 1980s (Al-Hussami *et al.*, 2017; Ayeleke *et al.*, 2018). In addition, factors such as rapid advancement in medical and information technology (Guo and Anderson, 2005), globalisation, changing demographics and an increasing number of elderly and people with chronic and composite diseases (Heinen *et al.*, 2019), scarce and diverse monetary and human resources (Ayeleke *et al.*, 2018), further increases the organisations’ complexity. This complexity makes healthcare systems challenging to manage (Baker and Denis, 2011; Jaafaripooyan *et al.*, 2020). Competent leadership is recognised as decisive for implementing reforms in health services (Ayeleke *et al.*, 2018), and the acquisition, retention and development of leadership competencies have been seen in conjunction with increased quality of services, higher efficiency and effectivity, and better performance and results in healthcare organisations (Groves, 2011; Trossman, 2011). As adequate leadership and managerial competencies are crucial to achieve the organisation’s goal, to deliver timely and high-quality services to the citizens (Kruk *et al.*, 2018), insufficient leadership can result in increased costs, reduced efficiency and effectiveness, lack of motivation, reduced job satisfaction and morale and ultimately dissatisfaction of patients as well as employees (Sfantou *et al.*, 2017). Competent leadership is therefore ubiquitous at all levels in the health sector (Stoller, 2017), and identifying the competencies aligned to effective leadership is the cornerstone for leadership development (McClelland, 1973).

As one of the first proponents of the term competence, McClelland defined competence as the “underlying characteristics of an individual, which is causally related to effective or superior performance in a job, role, or situation” (McClelland, 1973). Boyatzis (1982) later defined it as a capability or ability, while in conjunction with this understanding of the concept the KSA approach, entailing knowledge, skills and abilities, gained wide-ranging consensus the following decades. From the 1990s however, the definitions and descriptions of competence have broadened considerably in conjunction with an increased popularity of the concept. Terms such as *attributes, capacities, dispositions, attitudes* and *values* (Ellis, 1988) have been added, as well as individual characteristics (Lifrak *et al.*, 1997), personal traits (Veres *et al.*, 1990), personal- and task-oriented skills (Martina *et al.*, 2012) and experience (Lišková and Tomšík, 2013).

Despite the wide ranged application of the term, less effort has been made to define the concept (Mooney, 2007, p. 110). Emphasised by Françoise Delamare Le Deist and Jonathan Winterton (2005), there is such confusion and debate concerning the concept of “competence” that it is impossible to identify or impute a coherent theory or to arrive at a definition capable of accommodating and reconciling all the different ways the term is used (p. 29). The challenge with such lack of consensus is that the users underestimate the complexity of the concept and are in peril of defining and applying the concept through fundamentally different interpretations. Competence is said to be highly context specific (Brownell, 2008), leading to an excessive effort to develop one-dimensional frameworks that assert core competencies and associated descriptions thereof developed for specific contexts (Gunawan and Aungsuroch, 2017; Kantanen *et al.*, 2015; Liang *et al.*, 2020a, 2020b). Le Deist and Winterton (2005), on the other hand, argued that one-dimensional frameworks of competence are insufficient, and that a holistic framework might be a more adequate approach to identify the combination of competencies necessary for occupations (p. 1). A multi-dimensional holistic approach to healthcare leadership competencies can contribute to a common understanding of the concept as well as be applicable to several contexts and educational backgrounds, as it enables the users to identify combinations of competencies needed for leadership positions in health care that includes several perspectives on leadership competences. The main objective of this study is therefore to contribute to the field of research as well as the practise field by proposing a new understanding of what constitutes healthcare services leadership competence and to suggest a holistic framework for healthcare leadership competence that can be applied and developed further by both researchers, decision-makers and practitioners. Such framework can be used to identify specific leadership competency needs in healthcare organisations, to develop strategic competency plans and educational programmes.

## Aims

To achieve its objective, this study aims to answer the following two research questions:

RQ1. How are leadership competencies in the context of healthcare services described in empirical research articles, and what do they entail?

RQ2. How can leadership competence in the context of healthcare services be understood and operationalised holistically?

To answer these questions, a critical interpretive synthesis (CIS) was conducted. A CIS has previously been used to address complex issues in health care, such as access to health care (Annandale *et al.*, 2007; Woods *et al.*, 2005), cultural competence (George, 2015; Liu *et al.*, 2021), defining and classifying public health systems (Jarvis *et al.*, 2020) and implementation of evidence informed policies (Bullock *et al.*, 2021). To answer both research questions, competency descriptions retrieved from the included papers was coded, and new categories of healthcare leadership competencies developed. An analysis of the categories was conducted to develop a holistic understanding of leadership competence. To our knowledge, such multi-dimensional or holistic understanding of competence has not been developed for healthcare services leadership.

## Method: critical interpretive synthesis

### Study design

Given the heterogeneous nature of the term competence as well as the complex and divergent literature covering leadership competence in the health service, a traditional systematic review was neither a doable nor suitable approach for this study (Grant and Booth, 2009; Newman and Gough, 2020). Developed by Dixon-Woods *et al.* (2005) as a relatively new methodology with its offspring from the meta-ethnography tradition, a CIS is suitable to examine fuzzy concepts such as “competence” (Dixon-Woods *et al.*, 2006). In addition to producing a summary of the literature, a CIS methodology interprets the evidence, conducts appraisal and critique of the included papers and aims to produce a theoretical output in the form of synthesising argument (Woods *et al.*, 2005). To support the aim of theory generation, CIS uses some of the principles of grounded theory (Newman and Gough, 2020). As opposed to a traditional systematic review, this method aims for a comprehensive rather than exhaustive search of the literature (Dixon-Woods *et al.*, 2006). Aiming to generate a new understanding of leadership competence in the healthcare services, this study can be characterised as a configuring review; aiming to understand the phenomenon under investigation by interpreting existing descriptions of competencies to develop new concepts (Voils *et al.*, 2008).

## Search strategy

The search was conducted in two separate phases in March 2021 and August 2022 for the time January 2010–January 2022 using the databases Web of Science and CINAHL. Studies were limited to date and language, but not limited to study design. As it was the phenomenon leadership *competence* that was to be subjected to critical interpretation, synonyms such as skills, abilities and attributes was not included in the search. The final search string was AB = “manage* competenc*” OR AB = “leader* competenc*” AND AB = health sector OR AB = health service OR AB = health care OR AB = healthcare.

## Study selection

Papers were excluded based on titles and abstracts if they (1) did not have leadership competences in the health service as main objective, (2) did not give descriptions, characteristics and/or identification of leadership- or management competencies in the health service, (3) addressed education or development of competencies, (4) were not peer reviewed empirical studies or literature reviews and (5) were composed in other languages than English. The inclusion of papers was not restricted by profession, sector or country, as a general picture of how leadership competencies in the health service are described was the main objective. In accordance with the inductive approaches used in a CIS methodology, papers that met some of the inclusion criteria but not all were included in the early stages of analysis. This was also a consequence of the premise that it can be challenging to assess whether a paper would give actual descriptions of the competences or not based solely on the title and abstract.

## Data analysis

Inspired by Strauss and Corbin (1990), three phases of coding, namely, open-, axial- and selective coding, were conducted. The three steps of coding were completed repeatedly, moving back and forth between the steps. During the open coding, approximately 2,500 statements and keywords describing leadership competencies for the health sector were extracted from the data material and coded. This was a tedious process where each statement was discussed addressing the compass question: What knowledge, skills and abilities does this competency-description entail? The descriptions of the competencies were separated from the original code to avoid the original conceptualisation influencing the generation of a new categorisation. The axial coding consisted of putting the data back together in categories, where the coded material was reinterpreted. The main objective of this categorisation was to address what the competency descriptions comprising the concepts entailed. For example, the concept *strategy* entailed descriptions of strategic and analytical thinking which was categorised as cognitive abilities. Descriptions of ability to communicate the organisation’s strategy and vision to the staff and motivate them to work in accordance with the strategy were categorised as influencing and motivating staff. To prepare and implement strategy on the other hand was categorised as skills and abilities that described the steering part of leadership. A full table of the competence categories with subcategories and examples of descriptions can be found in the supplementary material, and a short overview is provided in the result section below. Following each step, the categories were systematically collated, compared and refined (Strauss, 1990). Near 60 pages of competency descriptions were cut out manually and sorted in new categories. After four rounds of coding, 12 categories with sub-categories were developed and as they were not challenged significantly in the fifth round, a satisfactory saturation was reached. The categories were then abstracted to produce a holistic understanding of what healthcare leadership competencies implies, by identifying a storyline of the phenomenon described in the material.

## Results

### Search results

**Figure 1.**
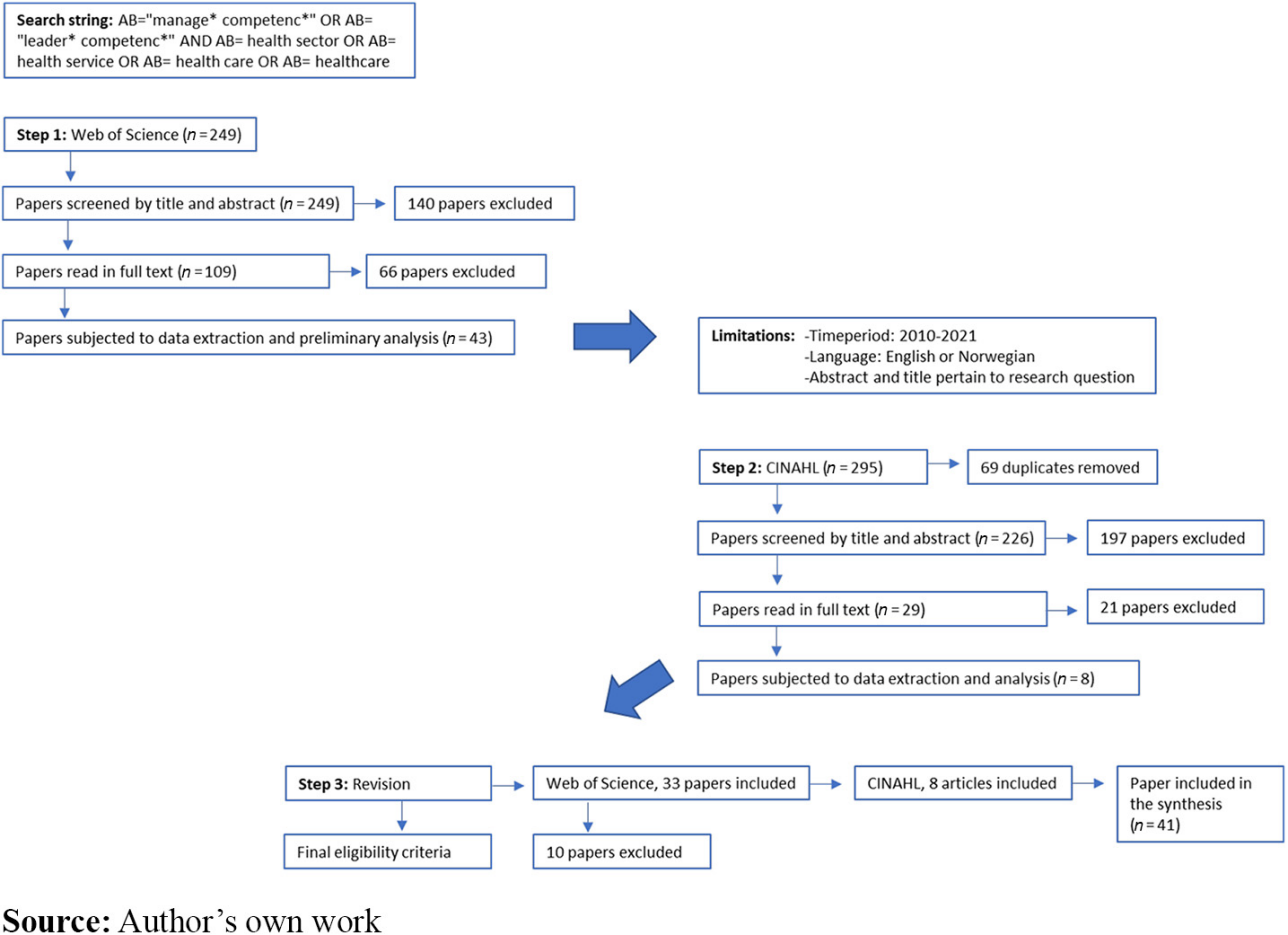
Process of study selection and search results

Electronic database searches resulted in 544 hits. After reading headlines and abstracts, 138 papers were read in full text and 41 papers were included in the study. A flow diagram outlining the search strategy and results is depicted in Figure 1. The first step started with a search in the database Web of Science in March 2021, which was concluded in June the same year. This resulted in 249 papers. Forty-three papers were subjected to data extraction and analysis. The second step started in January 2022 using a similar search strategy in the database CINAHL that was concluded in August the same year. One reviewer conducted the same procedure as in Step 1, resulting in eight included papers. In case of doubt, the other reviewer was consulted. All included papers from both searches were then read thoroughly again, resulting in 10 excluded articles from the first search due to lack of explicitly describing the competencies or not investigating leadership of professionals.

Of the 41 included papers, 31 were published in 2016 or later. Fourteen papers used quantitative methods, while 11 had a qualitative design, 11 used mixed methods and 5 were literature reviews. There was an uneven distribution in the included papers when it came to sector investigated: 19 papers examined hospitals, as opposed to only two examining primary care centres and six named primary health care as their main objective. Thirteen papers did not mention a particular sector. Most papers that investigated a certain profession studied nurses. Seventeen papers investigated nurse leaders, six studied physicians, three addressed several professions, and 15 papers did not specify any professional discipline. Of the 41 included papers, 18 based their empirical studies on an established competency framework or theory of leadership competency. A full overview of the included papers can be found in Appendix.

## Review of the literature

The data analysed in this study included several professions, public and private healthcare organisations, primary health care as well as hospitals, leadership levels from first line managers to senior leaders, and were conducted in 22 different countries on 6 continents. A systematic comparative analysis to investigate contextual differences in competence descriptions between countries, leadership level or profession was not conducted. During the coding of the descriptions of competencies the similarities were however striking as a clearer distinction between contexts was expected. There seem to be an agreement across papers regarding several key competencies such as communication skills, financial and business abilities, teamwork skills, knowledge of the health care and organisation, and professional knowledge, ethics and values amongst others. There are, however, some variations in the descriptions of the competencies. For example, all included papers except three explicitly named communication as an important leadership competency in the health service. Still, some describe communication as writing newspapers articles (Dikic *et al.*, 2020), while others describe it as listening and emphasising skills (Kakemam *et al.*, 2021) or preparation and delivery of business communications (Lopes *et al.*, 2020). According to the results from this study, these descriptions entail different kinds of skills and abilities and were therefore coded and placed in different categories.

## 12 Categories of healthcare leadership competence

Twelve different competence categories were developed, all separate and distinct. Some authors accentuate that the categories should be mutually exclusive (Patton, 1990), but even though there are clear distinctions between the categories, they do not meet the criteria of mutually exclusiveness. This is due to the descriptions that comprise each category can entail several aspects of knowledge, skills and abilities:

- *Character*: Involves competencies that are tied to personal skills and abilities, such as individual cognitive ability, personality and manners. This category has five sub-categories. (1) Includes self-awareness and be aware of own strengths and weaknesses. (2) Involves to control and manage own emotions, as well as understanding others’ emotions, behaviour and attitudes. (3) Are personal characteristics such as to work hard, solve problems and use good judgements. (4) Are characteristics such as being genuine and creative, have integrity and make decisions under extreme ambiguity. (5) Are mental abilities and cognitive skills such as abilities for critical and analytical thinking as well as problem-solving.
- *Interpersonal relations*: Entails skills and abilities to build and maintain relations with others, such as cooperation and understanding others’ needs and emotions. Four sub-categories were detected. (1) To create functional collaboration and teamwork, relating and working well with people, and interprofessional collaboration. (2) Abilities to develop interpersonal and effective relationships, establish mutual trust and respect and demonstrate listening skills. (3) To facilitate effective communication to enhance interpersonal relationships, to resolve communication barriers and to provide effective and constructive feedback. (4) To negotiate effectively and resolve conflicts, deal with difficult patients and their relatives and ability to reach consensus when discussing issues.
- *Leadership*: Entails competencies describing direct influence on others, such as being a mentor or a role model, to communicate the organisational vision and mission convincingly and influence the staff to believing in and wanting to achieve the organisational goals. Four sub-categories were identified. (1) Involves to influence subordinates, peers, and superiors in the realisation of organisational goals, and influence people to work together as a team. (2) Being a role model and a mentor, supporting and mentoring high potential staff, give advice and coach. (3) Includes descriptions of leadership actions that involve the staff directly in their work such as to delegate and assign tasks and responsibilities to staff based on their abilities, to realise the potential development of the work community and hold oneself and others accountable. (4) Involves skills and abilities to communicate organisational strategy, vision, mission and goal to the staff convincingly.
- *Professionalism:* Involves competencies specific to exemplary professional practice, professional development as well as values and professional ethics. Three sub-categories were detected: (1) Involves to work actively to develop, contribute to and enhance the professions, as well as own and staff’s professional knowledge, skills and abilities, ensure that the staff receive ongoing in-service training. (2) Includes ensuring ethical practise, that the right values are fostered and practised and to sustain a commitment to the community. (3) Contains skills, abilities and practices to be patient centred, improve patient care and quality of services, to go above and beyond expectations to focus on patients/patient care and developing quality assurance and improving patient safety.
- *Soft HRM*: Entails skills and abilities to organise staffs’ workday, to create a good work environment, and to arrange for staff’s development and education. This category contains four sub-categories. (1) To facilitate and take responsibility for the development, education and training of staff, provide a professional career ladder and focus on enhancing the skills of employees. (2) Facilitating the professional practice and welfare of the staff, to provide sufficient staffing, make flexible staffing plans and ensuring that staff are knowledgeable about what is expected from them. (3) Taking responsibility and action to create a safe and thriving work environment, monitor the work environment for potential safety issues and maintain a climate in which team members feel heard and safe. (4) Involves personnel management, recruitment and retention of personnel, and to use a supportive and collegiate management style.
- *Management*: Involves the steering part of leadership, such as directing and control, and target-oriented management, as well as implementing policy. Five sub-categories were identified. (1) To be result-oriented, to manage, ensure and evaluate performance and accomplishments, and to identify key criteria for performance evaluation. (2) To develop and implement strategies, visions and goals, setting the direction and to translate broad strategies into practical terms for others. (3) Includes resource allocation, financial management, business literacy, to mobilise processes to acquire resources and ability to use resources effectively and efficiently. (4) To ensure an efficient and effective organisation and improve processes, analysing the workflow of unit, identifying errors and ability to coordinate individuals and activities. (5) Includes administrative tasks such as establishing policy, systems and structures, and establishing rules and regulations, getting and administer human, financial, material and information aspects of the healthcare business.
- *Organisational knowledge*: Entails knowledge of own organisation, and ability to exploit internal knowledge in the purpose of organisational improvement. Includes four sub-categories. (1) Knowledge of own organisation’s rules and regulations, like the institution’s standard operating procedures, rules and the institution’s services. (2) Knowledge of the internal life and activities in the organisation, have organisational awareness and possess adequate knowledge of organisation issues. (3) Knowledge of how the organisation is structured and thereby how it functions, be able to effectively navigate organisational structures, roles and relationships and ability to create an understanding between working departments. (4) Involves abilities to coordinate operations, knowledge of operations, develop plans for operations in the organisation and knowledge of sequential and reciprocal task interdependencies.
- *Technology*: Includes skills and abilities to know of and be able to use information technology. This category has two sub-categories. (1) Involves to use and manage information technology, to use information technology in patient care management and delivery and practice, and be aware of ethical issues regarding information technology. (2) Development and improving services using new technologies and appreciate the need for development and modernisation of the system of communication to keep up with rapidly evolving technologies.
- *Knowledge of the healthcare environment*: Contains knowledge of how politics, other healthcare organisations, the community or municipality influence and affect own organisation. Three sub-categories comprise this category. (1) Involves abilities to envisaging potential impacts of decision making on operations, health care, human resources and quality of care, to implement plans and projects and to analyse and understand demographic, political, social, technical, cultural and economic factors and their impact on the organisation. (2) Understand how organisations in the healthcare sector interact and are interdependent, and to demonstrate understanding of the roles of key stakeholders in health care and how they interact. (3) Entails knowledge and understanding of the situation in the healthcare sector, how it works and identify development trends that can affect the organisation.
- *Change and innovation*: Includes to identify and respond to challenges and changes in the health service. Four sub-categories comprise this category. (1) Includes to lead and manage change successfully, to accommodate resistance, involving key stakeholders in designing and implementing change and practise shared decision-making in the process of change. (2) To identify the need for change and be able to analyse environmental developments that will necessitate change, have foresight and to anticipate resources needed to carry out initiatives. (3) Being able to initiate change to improve the organisation and services, like implementing improvement activities gradually over time, and be willing to challenge prevailing practice. (4) Taking initiative and facilitate for change and innovation, to be enthusiastic local “change agents”, and ability to use novel thinking in managerial planning.
- *Knowledge transformation*: Entails using knowledge from both internal and external sources. Competencies include to actively seek knowledge and information, to be enquiring and to learn outside of own context. This includes three sub-categories. (1) To facilitate for spreading knowledge to and among staff, to effectively share information and responsibility at different organisational levels and promote an openness to real interchange with those involved in the organisation. (2) To acquire knowledge from different sources, to use this knowledge in practice, produce knowledge, benefit from others’ experiences in the field of health and ability to collect and analyse valid information in an appropriate time. (3) To use knowledge and own and others’ experience, to implement best practice procurement, conduct evidence informed decision-making and to gather information to produce an evidence-based challenge to systems and processes to identify opportunities for service improvements.
- *Boundary spanning*: Involves competencies to coordinate and cooperate beyond the boundaries of own organisation. Three sub-categories constitute this category. (1) Includes creating and maintaining personal and organisational networks regionally, nationally and internationally, ability to understand stakeholder need and to maintain effective stakeholder relationships. (2) Involves to collaborate and build cooperative relationships with stakeholders or other organisations, to communicate and interact with external organisations and build constructive collaboration between new divisions and across agencies. (3) Includes being able to communicate to the environment through media or other channels and provide necessary information about the organisation to stakeholders, other organisations and the community.

## Discussion

The objective of this study was to contribute to the field of research as well as the practise field of healthcare services leadership with a new understanding of what constitutes healthcare services leadership competencies and to suggest a holistic framework for healthcare leadership competences that can be applied by both researchers, decision-makers and practitioners for further theoretical and practical development. To answer the research questions, a CIS of the literature was conducted to gather descriptions of healthcare leadership competencies. The result of the literature search provided a rich material to be analysed. Despite including papers from different contexts such as countries, organisations, professions and leadership levels, as well as a diversity in study designs, the retrieved descriptions of competencies resembled. Given that competencies are stated to be highly contextual (Brownell, 2008; Pihlainen *et al.*, 2016), this seems to be contradicted by the similarities in the descriptions across contexts found in this study. Several competency frameworks developed in one context were also confirmed in seemingly highly different contexts. This does not mean that healthcare leadership competencies are not contextual. It may however be an illustration of how challenging it is to identify specific competencies when dealing with such a broad concept in the context of the complex healthcare sector. The fact that many of the reviewed studies used competency frameworks developed in other countries may have contributed to concealing actual differences in competency needs. The competencies described in the material were also quite vague. For example, stating communication as a key competency for healthcare services leadership do not offer any guidance for practitioners as the descriptions given of this competency entail a wide range of highly different skills and abilities. This may therefore elucidate that one-dimensional frameworks for healthcare leadership competencies are not an adequate approach dealing with such a complex phenomenon, especially not if it is meant to be used across contexts. For example, if a healthcare leader wishes to apply research on healthcare leadership competencies to develop competencies in own organisation, it would be an insurmountable task to navigate the literature and get guidance on how to operationalise the frameworks to meet the needs of own organisation. Based on the review of the literature it can therefore be argued that a multi-dimensional holistic framework can be a more suitable approach. To try to accommodate this challenge in practise, this study aimed to develop a common understanding of healthcare leadership competencies based on what knowledge, skills and abilities the research studies describe them as. This resulted in 12 competence categories that describe healthcare leadership competencies and a suggestion of a tentative holistic framework for understanding the concept.

## Development of healthcare leadership competence categories

To answer *RQ1*, how leadership competencies in the context of healthcare services are described in empirical research articles and what they entail, descriptions of competencies were coded and categorised resulting in 12 competence categories. Although the amount of support for each category in the data differs, each category is assessed to have sufficient data to support them and it would make the category too diverse if it was to be merged into another category (Braun and Clarke, 2006). It has however been a question of discussion during analysis whether the categories with the lowest consensus in the included literature should and could be merged into other categories. But as this analysis was guided by the compass question “what knowledge, skills and ability do this competency description entail?” the eventual merge created too much diversity and are not in line with the inclusion/exclusion criteria of the existing categories. It is also a fair point that the included literature in this study is not exhaustive, hence there are probably leadership competency descriptions missing in the analysed data that could strengthen some of the categories or even contradict some. The categories presented in this study serve as a basis for further development and refinement. In addition to possible deficiencies in the sample, other significant changes in the environment can affect the competencies needed for healthcare leaders.

The competency descriptions were coded and categorised in several rounds that resulted in a continuous rearrangement of where the descriptions were placed. Several of the descriptions can also be interpreted to fit more than one category. For example, the description “interprofessional collaboration with trust, respect and ethical manner” (Gunawan *et al.*, 2019) entails skills and abilities suited for several categories. *Interprofessional collaboration* is prominent in (4) professionalism where interprofessional collaboration is interpreted as a part of developing and facilitating for professional practice. *Trust* is included in (1) character; to act and be trustworthy, and able to gain trust, (2) interpersonal relations; to be able to build trust and collaborative relationships, and (12) boundary spanning; ability to maintaining mutual understanding and trust with clients, communities, and other team members through effective communication. Whereas *ethical manner* is coded in (1) character; behaving in an open, honest and ethical manner, and (2) interpersonal relations; interprofessional collaboration with trust, respect and ethical manner, most descriptions involving ethics are coded in (4) professionalism where ethics and values is a sub-category. For practical implications, to identify specific competencies needed for successful interprofessional collaboration, these will be found in several of the categories.

## Suggesting a holistic framework to understand and operationalise healthcare leadership competence

**Figure 2.**
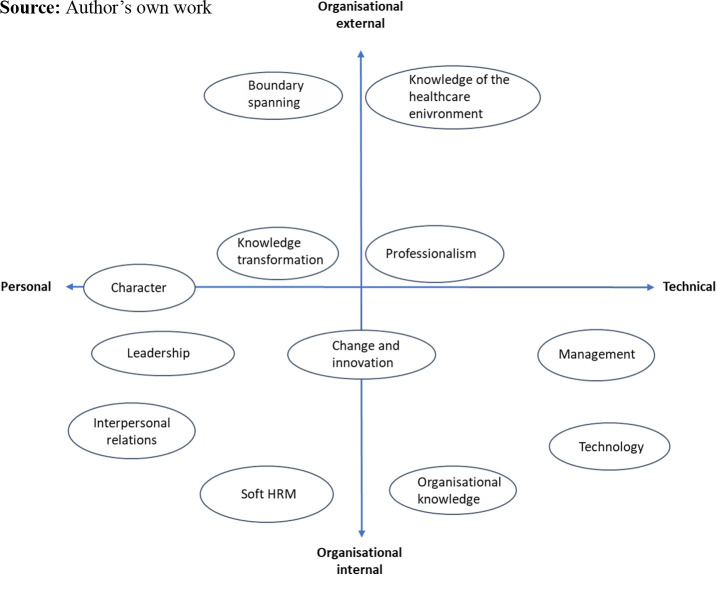
A holistic framework of healthcare leadership competence

*RQ2* asked for how leadership competence in the context of healthcare services can be understood and operationalised holistically. This was answered by an inductive analysis technique. During the selective coding, the content of the 12 categories was analysed at a higher level of abstraction that resulted in some central overall characteristics of the categories. As the categories were compared to each other, these abbreviated characteristics were identified as personal-, technical-, organisation internal- and organisation-external competencies. The personal competencies are personal characteristics, interpersonal skills and abilities, and skills and abilities to influence and motivate others. Technical competencies are knowledge, skills and abilities that enable a leader to conduct tasks such as clinical and professional abilities, budgetary skills, strategy development or ability to apply information technology. Some of the competencies were relevant for practicing within the organisation, while others were tied to the external environment of the organisation. For example, descriptions like “building constructive collaboration between new divisions and across agencies” were placed in (12) boundary spanning with emphasis on organisational external competencies, and not in (2) interpersonal where most of the descriptions including collaboration were placed. This way of interpreting the categories also made the distinction between, for example, (6) management and (3) leadership clear, where (6) management is comprised of technical skills tied to the steering part of leadership (e.g. establishing policy, systems and structures), and (3) leadership consists of personal skills to influence and motivate staff (e.g. create and communicate a shared vision for the future and inspire team members to achieve it). The categories are however not exclusively personal, technical, organisational internal or -external, but can be interpreted as dimensions along those axes. This is depicted in Figure 2.

Figure 2 illustrates that the 12 competence categories can be predominantly personal (1. character, 3. leadership, 2. interpersonal relations), predominantly technical (6. management, 8. technology) or have an even distribution of personal and technical competencies (12. boundary spanning, 9. knowledge of the healthcare environment, 11. knowledge transformation, 4. professionalism, 5. soft HRM, 7. organisational knowledge). They can be predominantly tied to the organisation (2. interpersonal relations, 3. leadership, 5. soft HRM, 10. change and innovation, 7. organisational knowledge, 6. management, 8. technology), predominantly tied to the external environment of the organisation (12. boundary spanning, 9. knowledge of the healthcare environment) or be evenly tied to both internal and external the organisation (4. professionalism, 11. knowledge transformation). The competence categories include multiple competency types, such as behavioural, functional, personal and cognitive competence.

For practical utilisation of the holistic framework suggested in this study, one needs to map the competency requirements in the unit, department or organisation in question. At different leadership levels as well as in different contexts, the emphasis of each category will differ and all may not be relevant at all. The degree of technical versus personal, internal or external can also vary depending on which competencies are needed.

## Strengths and limitations

This literature review was conducted with results from only two databases. In this study, identifying the right leadership competencies was not an aim, but rather to establish a general picture of how competencies in the context of health care are described in empirical research articles. Therefore, a quite wide net was cast using a search string that allowed hits for all peer-reviewed research articles published from 2010 until 2022 that included leadership or management competence in health care. Because it was the term competence that was under investigation in this study, synonyms such as skills, abilities, characteristics, knowledge and other terms alike were excluded. Because of this, it is likely that there exist empirical studies that could have added to the development of the competence categories or could have contradicted some of the descriptions used in this analysis that were assessed as quite harmonised. A CIS also opens for a broader search, including grey literature, books and policy documents, but this study was limited to published empirical studies. This limitation could also have affected the result.

The results of this study indicate that the descriptions of healthcare services leadership competencies are somewhat general and contradictory to the statement that competences are highly contextual. This might also be a result of the search string used in this study that was shaped in a general manner excluding synonyms of the comprehensive term competence. Conducting searches investigating professions, sectors and leadership levels separately could have revealed clearer contextual differences than this study intercepted. In other words, a generic search for leadership competencies in the health service might have led to a generic result of descriptions of such. The 12 competence categories developed in this study must therefore be considered indicative in line with the inductive and explorative approach in this review and as a consequence of the limitations in the search strategy. The strength of this study is however that the data retrieved from the included papers was exposed to a thorough and iterative coding and analysing process guided by grounded theory. The suggested framework represents a new understanding of how both researchers and practitioners can operationalise healthcare services competencies.

## Conclusions

This study is an effort to tentatively suggest a holistic framework for healthcare services leadership competencies to clarify the overarching usage of the concept. This study suggests 12 competence categories that offer a new understanding of healthcare leadership competencies. This framework can support a unifying understanding of how healthcare services competencies can be understood, contributing to a common language handling a concept filled with diversity of views and making it possible to operationalise the competency needs in a healthcare organisation.

## Supplementary Material



## Figures and Tables

**Table A1.** Overview of included papers

First author	Year	Title	Methodology; aim of study	Participants	Country	Profession/ Sector	Level	Framework
1. Kakemam, Edris	2021	Developing competent public hospital managers: a qualitative study from Iran	Qualitative research design included position description analysis and focus group discussions; identifying managerial competencies required by middle and senior-level managers in the public hospitals	PD = 58 FGD = 19 tot.; 9 senior and 10 middle managers	Iran	Hospital	Senior and middle managers	MCAP [1]
2. Garman, Andrew N.	2020	Bridging worldviews: toward a common model of leadership across the health professions	Mixed methods including four phases: future scan, behavioural event interview data, electronic survey and natural language processing; To revise and revalidate a widely used health sector leadership competency model and assess its potential for providing greater interoperability across the professions	Survey: *n* = 148	USA	Hospital, health system or clinical practice	Chief-level executive, Leader of managers, Manager, Direct contributor	NCHL [2]
3. Pounder, Paul	2019	Impassioned leadership effectiveness: an assessment of leadership styles of top leaders in Caribbean healthcare systems	Grounded theories as the basis for thematic analysis; explore effective leadership based on information collected from leaders in the healthcare delivery system within the Caribbean;	20 officials	Caribbean	n/a	Ministers, permanent secretaries, and chief medical officers from ministries of health	n/a
4. Fanelli, Simone	2020	Managerial competences in public organisations: the healthcare professionals’ perspective	Mixed methods, three phases: literature review, focus groups, and questionnaire; to identify specific managerial competences perceived as crucial by healthcare professionals	Questionary: *n* = 585	Italy	Healthcare workers, physicians, nurses, veterinarians, psychologists in public healthcare organisations	Managers and professionals	n/a
5. Dikic, Milica	2019	Alignment of perceived competencies and perceived job tasks among primary care managers	Quantitative, CDC-US structured questionnaire; to explore how managers in primary health care organizations assess their managerial knowledge and skills, as well as the importance of these competencies	106 respondents	Serbia	Primary care centres	Directors, deputy directors, department heads, head nurses, and lower managerial positions	CDC-US [3]
6. Walsh, Aidan P.	2019	Are hospital managers ready for value-based healthcare?	A systematic literature review to identify research studies that describe the characteristics of management competence in hospital environments	22 articles	n/a	Hospitals	n/a	n/a
7. Ofei, Adelaide M. A.	2020	Exploring the management competencies of nurse managers in the Greater Accra Region, Ghana	A quantitative exploratory design was used to assess whether nurse managers has all the management competencies of the Katz model	552 nurses	Ghana	Nurse, hospitals, primary health care	Unit level	Bristow (2001) and Katz (1974)[4]
8. Kakemam, Edris	2020	Leadership and management competencies for hospital managers: a systematic review and best-fit framework synthesis	A systematic literature review; to synthesize the evidence related to the leadership and management competencies in healthcare organizations	12 articles	n/a	Hospital	n/a	MCAP
9. Lopes, Alipio Gusmão	2019	An assessment of management competencies for primary health care managers in Timor-Leste	A cross-sectional survey; to assess the levels of management competencies of primary health care	Questionnaire: 183 PHC managers	Timor-Leste	Primary health care	Upper level	n/a
10. Heinen, Maud	2019	An integrative review of leadership competencies and attributes in advanced nursing practice	An integrative review; to establish what leadership competencies are expected of master level‐educated nurses	15 articles and 7 frameworks	n/a	Nurse (advanced practice nurses and clinical nurse leaders)	n/a	n/a
11. Pihlainen, Vuokko	2019	Experts’ perceptions of management and leadership competence in Finnish hospitals in 2030	A three-round, Web-based Argument Delphi process; to elicit and analyse experts’ perceptions of management and leadership competence (MLC) and likely MLC developments and requirements in hospital contexts by 2030	33 Finnish leadership and management experts; 31 out of 33 recruited panellists participated in the first round; 27 in the second round and 25 in the third round	Finland	Hospital	n/a	n/a
12. Dorji, Kinley	2019	Leadership and management competencies required for Bhutanese primary health care managers in reforming the district health system	A quantitative method with a cross-sectional survey; to identify the required management competencies, current competency levels, and strategies for improving the management competencies	339 PHC managers	Bhutan	Primary health care	Multiple levels	n/a
13. Gulati, Kamal	2019	Medical leadership competencies: A comparative study of physicians in public and private sector hospitals in India	A survey questionnaire; to evaluate medical leadership competencies of public and private sector doctors	532 doctors	India	Private and public sector hospitals, doctors	n/a	MLCF [5]
14. Yakubu, Kenneth	2019	A comparison of leadership competencies among doctors practicing in public and private hospitals in Jos Metropolis of Plateau State, Nigeria	Cross-sectional, comparative multicentre study including self- and peer assessment; to assess and compare perceived leadership competencies of doctors occupying managerial positions	27 doctors (89 health and non-health professionals)	Nigeria	Private and public hospitals, doctors	CMD/CEO HOD/Unit Head	NCHL
15. Liang, Zhanming	2018	An evidence-based approach to understanding the competency development needs of the health service management workforce in Australia	360° assessment of the competence; to identify managerial competence levels, and training and development needs	93 health service managers	Australia	Hospital	Mid-level	MCAP
16. Amini, Zahra	2018	Identifying social entrepreneurship competencies of managers in social entrepreneurship organizations in healthcare sector	Qualitative interviews; to identify the social entrepreneurship competencies of managers in social entrepreneurship organizations in the field of health care	8 managers	Iran	n/a	n/a	n/a
17. Nazari, Roghieh	2018	The meaning of managerial competency of ICU head nurses in Iran: a phenomenological study	Qualitative approach, extracted the lived experience of ten Iranian ICU head nurses; to explore the meaning of managerial competence of head nurses in intensive care units (ICU) in Iran	10 ICU head nurses	Iran	Hospital, nurses	Unit head nurse	n/a
18. Ireri, Salome Kathomi	2017	A comparison of experiences, competencies and development needs of doctor managers in Kenya and the United Kingdom (UK)	A comparative study design involving fieldwork, qualitative interviews and a survey; to explore and compare the experiences, leadership and management competencies and development needs of doctor managers	Interview: 25 from NHS organisations Survey: 302	Kenya and UK	Doctor managers, public and private. Clinical directors from NHS and hospitals.	Director, director assistant	MLCF
19. Kantanen, Kati	2017	Leadership and management competencies of head nurses and directors of nursing in Finnish social and health care	Quantitative questionnaire; to describe leadership and management competencies of head nurses and directors of nursing	1025 nurses	Finland	Specialised and primary health care, and social care, nurses	Senior, middle and unit/ward level	NMLMC [6]
20. Gunawan, Joko	2017	Managerial competence of first‐line nurse managers: a concept analysis	A concept analysis; to clarify what is meant by managerial competence of first‐line nurse managers internationally, what attributes signify it, and what its antecedents and consequences are	8 articles (managerial competence)	n/a	Nurse	First-line	n/a
21. Hargett, Charles W.	2017	Developing a model for effective leadership in healthcare: a concept mapping approach	Literature review, focus groups, and consensus meetings; to identify stakeholders’ mental model of effective healthcare leadership, clarifying the underlying structure and importance of leadership competencies	Focus group: 19 Card sorting: 92	USA	Physicians	n/a	DHLM [7]
22. Li, Wenqin	2016	A study of leadership competencies of first-line nurse managers in Shanghai, China using Delphi technique	The Delphi technique; to explore leadership competencies needed by first-line nurse managers	Expert panel: 20	China	First-line nurse managers	First-line	n/a
23. Munyewende, Pascalia O.	2016	An evaluation of the competencies of primary health care clinic nursing managers in two South African provinces	A cross-sectional study; to evaluate the competencies of PHC clinic nursing managers	105 nursing managers	South Africa	Primary health care, nurse	Clinic nursing managers	n/a
24. Murphy, Kelly R	2016	Design, implementation, and demographic differences of HEAL: a self-report health care leadership instrument	A 24-item survey was designed; to measure leadership competency based on the core competencies and core principles of the Duke Healthcare Leadership Model	Pilot survey: 126 health care professionals Focus group: ∼40 physician medical leaders	USA	Physicians, physician assistants, and medical students	Various levels	DHLM
25. Pihlainen, Vuokko	2016	Management and leadership competence in hospitals: a systematic literature review	Systematic literature review; to describe the characteristics of management and leadership competence of healthcare leaders and managers	13 papers	n/a	Hospitals; nurses and physicians	n/a	n/a
26. Kitreerawutiwong, Keerati	2015	Development of the competency scale for primary care managers in Thailand: scale development	In-depth interviews and focus group discussions, and factor analysis; to develop and examine the psychometric properties of the competency scale for primary care managers	Interview and focus group: 35 Survey: 487	Thailand	Primary care	n/a	n/a
27. Kantanen, Kati	2015	The development and pilot of an instrument for measuring nurse managers’ leadership and management competencies	Literature review, expert panel and survey; to develop and piloting of an instrument for measuring nurse managers’ leadership and management competencies	Test survey: 22 (nursing managers) Expert panel: 23 (doctoral students in nursing science)	Finland	Nurses	n/a	NMLMC developed
28. Kovacic, Helena	2015	Leadership competences in Slovenian health care	Survey; to examine leadership competences of managers in the healthcare sector	265 employees in health care and267 business managers	Slovenia	Health professionals or nursing professionals	n/a	Mintzberg[8]
29. Martins, Jo M.	2015	An evidence-based framework: competencies and skills for managers in Australian health services	Evidence-based approach, Analysis of the salient systemic changes; to provide evidence from the real world to identify competencies/ skills that will enhance the performance of health service managers.	n/a	Australia	n/a	n/a	n/a
30. Hopkins, Margaret M.	2015	Distinguishing competencies of effective physician leaders	Critical incident interviews; to determine the particular competencies demonstrated by effective physician leaders.	28 physicians	USA	Physician	n/a	ECI [9]
31. Kvas, Andreja	2013	The use of competency models to assess leadership in nursing	Survey; to develop a competency model for leaders in nursing, and to compare it with the leadership competency model for state administration.	141 nurse leaders	Slovenia	Nurse, hospital	First level (Head nurses in hospitals and clinics) Second level (ward managers and section managers) Third level (leaders of small units and teams)	n/a
32. Supamanee, Treeyaphan	2011	Preliminary clinical nursing leadership competency model: a qualitative study from Thailand	Qualitative in-depth interviews and focus groups; to explore the clinical nursing leadership competency perspectives of Thai nurses	In-depth interviews: 23 nurse administrators Focus groups: 31 registered nurses	Thailand	Nurse, hospital	Head nurses, supervisors, heads of nursing sections	n/a
33. Aitken, Kim	2013	Organisational and leadership competencies for successful service integration	Literature reviews and semi-structured interviews; to identify the key organisational and leadership competencies required to ensure successful service integration within a coalition framework	Interviews regarding leadership competencies: 7 key managers from the consortia	Australia	n/a	Consortum centres leaders	n/a
34. Ángel-Jiménez, Gloria María	2013	Relevance level of application of management competencies in nursing	Survey and semi-structured interview; to identify the relevance and level of application of the main management competencies in nursing	Survey: 140 individuals from the nursing faculties Interviews: 6 experts from the university educational sector	Colombia	Nurse, private and public sector	Multiple levels	n/a
35. Czabanowska, Katarzyna	2013	In search for a public health leadership competency framework to support leadership curriculum–a consensus study	A literature review, consensus development panel and Delphi survey; to develop a public health leadership competency framework to inform a leadership curriculum for public health professionals	Expert panel: 7 public health and 7 leadership academics from four European Universities Delphi survey: 10	Netherland, Europe	Public health	Senior public health professionals	n/a
36. González - García, Alberto	2021	Nurse managers’ competencies: a scoping review	A scoping review; to describe and synthesize scientific literature on nurse managers’ competencies	Studies included: 76	n/a	Nurse	n/a	n/a
37. Gunawan, Joko	2020	Comparison of managerial competence of Indonesian first-line nurse managers: a two-generational analysis	A cross-sectional survey in 18 public hospitals; to compare first-line nurse managers’ managerial competence according to generational analysis across public hospitals	Survey: 254 nurse managers	Indonesia	Nurse, public hospitals	First-line	I-FLNMMCS[10]
38. Gunawan, Joko	2020	Perceived managerial competence of first-line nurse managers: a comparative analysis among public hospitals	A cross-sectional study; to identify managerial competence of first-line nurse managers according to hospital type and ownership	Survey: 233 nurse - managers	Indonesia	Nurse, public hospitals	First-line	I-FLNMMCS
39. Gunawan, Joko	2019	Development and psychometric properties of managerial competence scale for first-line nurse managers in Indonesia	A survey based on integrative review and expert interviews; to develop and psychometrically test the managerial competence scale for first-line nurse managers	Survey: 300 nurse - managers	Indonesia	Nurse, public hospital	First-line	I-FLNMMCS
40. Liou, Yung-Fang	2021	Psychometric properties and development of the competency inventory for Taiwanese nurse managers across all levels	Mixed methods, including literature review, qualitative study for generating the preliminary inventory and a cross-sectional survey; to describe the development and psychometric testing of the competency inventory for nurse managers	Survey: 573 nurse - managers Interview: 5 stakeholders Expert panel: 5 experts	Taiwan	Nurse, hospital	Front-line and mid-level	Katz framework of managerial skills
41. Moghaddama, Nader Markazi	2019	Managerial competencies of head nurses: a model and assessment tool	Literature review, Delphi technique and expert panel; to provide a valid tool for assessing managerial competencies of hospital department head nurses	Expert panel 1: *n* = 16 Expert panel 2: *n* = 5 Panel discussion: *n* = 7 Expert panel 3: *n* = 10 Survey pilot: *n* = 30 Survey test: *n* = 20	Iran	Nurse, hospital	Department head nurse	n/a

**Source:** Author’s own work

## Notes

1. Management Competencies Assessment Tool, Australia, (Liang *et al.*, 2020).

2. National Center for Healthcare Leadership, USA, (Calhoun *et al.*, 2008; National Center for Healthcare Leadership. 2018).

3. Centers for Disease and Control in the United States, USA, https://stacks.cdc.gov/view/cdc/13701/cdc_13701_DS1.pdf

4. Bristow, M. (2001). Management competencies. MSc thesis, School of Management, UMIST Manchester; Katz, R. (1974). Skills of an effective administrator. Harvard Business Review, 31(1), 33–42.

5. Medical Leadership Competency Framework, National Health Services (NHS), UK (2010).

6. Nurse Managers’ Leadership and Management Competencies Scale, Kantanen *et al.* (2015).

7. Duke Healthcare Leadership Model.

8. Mintzberg H. *The nature of managerial work*. New York: Harper & Row, 1973.

9. Emotional Competence Inventory, Boyatzis *et al.* (2002).

10. Indonesian-First-Line Nurse Managers Managerial Competence Scale. Gunawan, J., Aungsuroch, Y., Fisher, M.L. and McDaniel, A.M. (2019). Development and psychometric properties of managerial competence scale for first-line nurse managers in Indonesia.

## Supplementary material

The supplementary material for this article can be found online.

## Appendix

Table A1
