# Supplementary material for: Suggesting a holistic framework for understanding healthcare services leadership competence – a critical interpretive synthesis
Source: Leadersh Health Serv (Bradf Engl). 2024 Apr 15;37(5):99–129. doi: 10.1108/LHS-08-2023-0059 (PMC11348838; doi:10.1108/LHS-08-2023-0059)
Supplement: Supplementary file 1 [file leadershhealthserv-37-0099-s001.docx]

**Categorisation of healthcare leadership competencies**

| 1. **Character**   This competence category involves individual characteristics such as thought patterns, cognitive abilities, personality, manners, and way of being. Some of the competence-descriptions give clear descriptions as “character”-competencies, such as “admits criticisms, use good judgment, stress management techniques, control and manage emotions appropriately, flexibility, time management techniques, self-assessment for awareness of personal strengths and weaknesses and empathy” (Kakemam et al., 2021), “continuously improve self-knowledge, interpersonal effectiveness and well-being” (Garman et al., 2020) or “being genuine, having integrity, a strong will to lead, strong self-belief, thinking outside the box and understanding own emotions” (Pounder & Greaves, 2020). In other cases, the character-competences are a premise to have other competences. For example the description of being “development oriented” and “willing to challenge prevailing practice” (Pihlainen et al., 2019) can be categorised as the competence type (10) change and innovation, but the personal characteristics necessary to fulfil the descriptions are also suited to character.  Five sub-categories were identified in the competence descriptions of this category. (1) *Self-development* comprise of descriptions regarding the ability to continuous developing own skills and abilities. It also entails descriptions such as self-confidence, self-awareness, self-belief, and self-management, as well as self-evaluation, being able to learn from experiences, improve self-knowledge and awareness of personal strengths and weaknesses. (2) *Emotional Intelligence* entails descriptions of skills and abilities in being able to control and manage own emotions, as well as understanding other’s emotions, motives, and desires. To see oneself and how others perceive you were also described, and being able “to read the room”. The third sub-category labelled (3) *do* was developed based on personal characteristics that results in actions. Examples of descriptions of this sub-category are to manage time; work hard; use good judgement; solve problems; accommodating cultural differences; adapt communication style to situation; and to ability to make decisions under extreme ambiguity. The fourth sub-category called (4) *be* entails characteristics of who you are as a person. Examples of competence descriptions are to possess a sense of humour; be flexible; have good listening skills; being genuine; truthful; creative; and decisive; have high levels of integrity and non-wavering values. (5) *Cognitive* involves mental skills and abilities. This includes thinking logically; analytical thinking; thinking outside the box; strategic thinking; problem solving abilities (that was differentiated from the action of solving problems in the sub-category “do”); abilities to think critically; novel and innovative thinking; and ability to rapidly acquire new knowledge. | | | | | | | | | | | | | | | | | | | | | | | | | |
| --- | --- | --- | --- | --- | --- | --- | --- | --- | --- | --- | --- | --- | --- | --- | --- | --- | --- | --- | --- | --- | --- | --- | --- | --- | --- |
| **Sub-categories of *character* with examples of descriptions:** | | | | | | | | | | | | | | | | | | | | | | | | | |
| **Self-development**  Admits criticisms  Self-assessment for awareness of Personal strengths and weaknesses  Ability to identify one's own strengths and weaknesses and make effort toward improvement  Ongoing habits and actions taken to continuously improve self-knowledge, interpersonal effectiveness and well-being  Self-Confidence    Strong self-belief  Self development  Self-knowledge  Self-evaluation  Developing self awareness  Being able to learn from experiences  Continuing personal development  Sustained personal commitment  Awareness of personal strengths and weaknesses  Managing Self  Cultivating Personal Resilience  Demonstrate the value of lifelong learning  Aware of own personal limitations  Personal development skills  Know how to receive criticism  Know how to develop myself  Accurate Self-Assessment  Be active learners who strived to have continuous self-development of themselves and other  Respond appropriately to the positive criticism of others about your own behaviour or performance  Self-direction  Learning from mistakes and making effort to correct them in an appropriate time  Managing Personal performance  Commitment to self-development  Being influenced by role model in the field of health | | | **Emotional Intelligence**  Control and manage emotions appropriately  Empathy  Recognising the value of emotional intelligence    Understanding of their feelings, motives and desires  Appreciation of who they are; and how others around them saw them  Emotional stability  Handle emotionally stressful situations appropriately  Emotional Self-Awareness  Emotional Self-Control  Control their emotions when they face serious problems or conflict situations  read “what is not being said”  Be aware of the impact of your own behaviours and reactions on the behaviours and reactions of others  Emotional intelligence  ability to identify and control emotions  Dominate behaviour and guide it towards a positive manner  Ability to control emotions and regulate behaviour  Demonstrate awareness of the impact of your own beliefs, values, and behaviours on your own decision-making and the reactions of others | | | | | | **Do**  Managing time, Time management, Time management techniques  Work hard, be responsible and achieve reliable and satisfactory performance  Use good judgment  Stress management techniques  Solving problem    Accommodating cultural differences  Displayed consistency of their actions  Uniformity in their methods  Showcase their will in their negotiation tactics  Recognise their capabilities and belief system to achieve required goals  Personal and professional balance  Takes initiatives  Responsiveness  Perseverance and pursuit  Balancing work and life issues  Maintaining Personal Balance  Organising and managing themselves while taking account of the needs and priorities of others  Behaving in an open, honest and ethical manner  Acting independently  Demonstrate maturity  Establish a good image that is spirited, amiable and elegant temperate  Act in a manner that is open, honest, and trustworthy  Act decisively  Communicate effectively with patients  Initiative  Display assertive leadership behaviors  “daring” to ask questions about doctors’ treatment orders,  Composing different opinions  Giving advice and suggestions  Demonstrate a range of effective communication skills  Ability to listen and consult  Adapt communication style to suit the needs of the situation and audience  Interact effectively with the [client group]  Well-developed written communication skills, including an ability to write cogent reports under time pressure  Demonstrates achievement focus and drive  Demonstrates tenacity and resilience  Demonstrate effective written and oral communication, and presentation skills  Recognise and use non-verbal forms of communication when putting across your own perspective on a situation  Share views in a non-judgmental, non-threatening way  Exercise the sensitivity needed to communicate with diverse cultures and disciplines.  Demonstrate personal responsibility and accountability for the achievement of a given task  Demonstrate resilience and the ability to call upon personal resources and energy at times of threat or challenge  Dealing with anger, criticism, and frustration in a constructive manner  Demonstrate Resilience: • recover from set-backs; and • discuss coping mechanisms  Making decisions in conditions of extreme ambiguity | | | | | | | **Be**  Remain calm and composed in emergency situations  Display appropriate sense of humor  Possesses a sense of humour  Personal mastery  Flexibility  Good listening skills    Being genuine  Being aware of own values, principles and assumptions  Truthfulness that demonstrate their deepest principles and values    High levels of integrity  Ability to get along with people  Patience to listen to people  High level of soundness and wholesomeness  Prioritize  Multi-task  Strong wills and a drive to lead  Persistence in working toward a series of goals and outcomes    Willingness to adapt  Trust, empathy and sympathy  Creativity  Internal locus of control  The physical and mental ability to work under difficult conditions  Forbearance  Changeability  Feeling committed  Confident appearance  Enthusiasm for learning  Acting with integrity  Resilience and composure  Integrity and ethical stance  Personal integrity  Being Decisive  Stay calm and remain optimistic under pressure and when facing misunderstanding  Be trustworthy and fair  Gaining trust  Know how to cope with stress and complex situations  Place the needs of the team above my own needs  Accept responsibility after undesirable outcomes  Adapt well to change  Transparency  Adaptability  Achievement focus  Optimism  Operates with integrity and professionalism  Is flexible and adaptable  Selflessness, integrity and perspective mastery  Credibility  Enthusiasm  Commitment  Honesty  Caring  Compassionate  Ability to gain employees’, customers’ and colleagues' trust  Ability to effectively work with individuals of diverse race, gender and culture  Serving selflessly  Work hard and be responsible | | | | | | | | | **Cognitive**  Thinking outside the box  Good analytical ability  Demonstrating critical and reflective thinking  Analytical thinking  Thinking logically  Organising the parts of a scenario systematically, comparing different features or aspects  Setting priorities rationally  Identifying time sequences,  casual relationships or if–then relationships  Methodical step-by-step approach to thinking that enables quick and effective problem-solving  Strategic thinking,  Creativity  Be innovative and creative  Abilities to think critically  Ability to rapidly acquire new knowledge  Innovative thinking  Possesses highly-developed critical thinking and decision-making skills  Strategic thought or understanding  Strategic thinking (a more creative, visionary, and reflective process that provides a broader and long-term view of the organization)  Circumspect and rapid thinking skills  Competencies to think carefully and swiftly |
| 1. **Interpersonal relations**   Contains descriptions of skills and abilities that enables a person to create and maintain interpersonal relations with others. The material described skills like listening, supporting others, gaining trust, showing understanding, working within teams (Ireri et al., 2017), building and maintaining relationships (Hargett et al., 2017; Kantanen et al., 2015, 2017; Pihlainen et al., 2016), collaborating interprofessionally (Gunawan & Aungsuroch, 2017), build trust and collaborative relationships with the members of the multidisciplinary team, listening skills (Li & Wivatvanit, 2016), relating to people and developing collaborative relationships within the organization (Pihlainen et al., 2016), as well as communicate effectively (González García et al., 2020) and giving and receiving effective feedback (Moghaddam et al., 2019).  The category interpersonal relations contains four sub-categories. A range of competences described relational skills and abilities necessary to create functional collaboration and teamwork and was coded as (1) *collaboration.* Examples include proficiency in relating and working well with people based on one’s knowledge about people, how they behave and operate in groups; skills in enhancing group dynamics; acknowledging, and appreciating efforts, contributions, and compromises; and interprofessional collaboration with trust, respect, and ethical manner. The second sub-category (2) *relationship* is composed of skills and abilities to manage, create, and maintain relationships. Examples are listening attentively to the concerns of others both within and outside the organisation; developing interpersonal and effective relationships; relationship building; establish mutual trust and respect; demonstrate listening skills; listening to workers’ concern; supporting others; ability to mobilise the capacities of others; and value and respect diversity. (3) *Communication* was one of the concepts where the descriptions were distributed to most of the categories. In relation to this category and sub-category involving interpersonal relation skills, the descriptions were for example: resolving communication barriers; provide effective and constructive feedback; determine the appropriate communication mode according to the situation; assess the internal climate, including the values, feelings, and temperament prior to communication; engage confidently and constructively in verbal and nonverbal interactions; and establishing effective interpersonal and inter-professional communication. (4) *Negotiation* is a sub-category that contains competence descriptions of skills and abilities to negotiate effectively and resolve conflicts. Descriptions like resolve conflicts among staff and managing group conflicts within the organisation; manage conflict professionally and actively; deal with difficult patients and their relatives; ability to reach consensus when discussing issues; and deal with difficult situations at the workplace were included. | | | | | | | | | | | | | | | | | | | | | | | | | |
| **Subcategories of *interpersonal relations* with examples of descriptions:** | | | | | | | | | | | | | | | | | | | | | | | | | |
| **Collaboration**  Proficiency in relating and working well with people based on one’s knowledge about people, how they behave and operate in groups  Teamwork  Collaboration  Team Leadership  Being a team member  Enhancing group dynamics  Leading, building and managing teams  Team building and collaboration  Collaborates with healthcare professionals, including physicians, advanced practice nurses, nurse managers, and others  Working within teams to deliver and improve services  Have a clear sense of their role, responsibilities and purpose within the team  Adopt a team approach  Acknowledging and appreciating efforts, contributions and compromises  Ability to deal with opposing viewpoints and guiding teamwork towards common goals of the organization  Collaborating interprofessionally  Optimizing team dynamics  Managing team performance  Development of collaborative relationships within the organisation  Build trust and collaborative relationships with the members of the multidisciplinary team  Engage staff in decision making  Effectively utilise the strengths of team members  Give credit to team members  Teamwork skills  Ability to be flexible when working with the team  Committed to collaborative planning at regular intervals  Interprofessional collaboration with trust, respect and ethical manner  Creating effective teamwork  Working as an effective team member  Spirit of teambuilding  Consult other employees when making strategic decision  Exhibits collective and collaborative abilities  Promoting group cohesion  Employs consultative and leadership skills with intraprofessional and interprofessional teams  Value shared decision-making  Spirit of teambuilding,engagement and inclusion | | | **Relationship**  Relationship management  listening and empathising with staff skill  Actively listen to others’ ideas, suggestions, and opinions  Listening with understanding  Interpersonal understanding  Inter-personal skills  Listen attentively to the concerns of others, both within and outside the organization  Value and respecting diversity  Develop interpersonal relationship  Demonstrating effective relations  Developing effective relationships and collaborations in support of organisational goals  Relationship building  Building and maintenance of interpersonal relationships  Being accepted by the personnel and the authorities  Building and maintaining relationships  Supporting others  Showing understanding  Develop rapport with a variety of people and make friends with subordinates  Care about nurses as individuals  Treat subordinates with respect in terms of their interests and achievements, and create an environment which recognise and value differences in others  Engage staff in decision making  Have a high level of moral and treat others politely  Relating to people  Listen to workers’ concerns  Professional colleagues rely on me as an authoritative source of advice.  Ability to mobilise the capacities of others.  Developing peer relationships  Human relationships  Develop good relationships with a variety of people and groups within the nursing and multidisciplinary teams, and with patients and families  Establishing mutual trust and respect by dealing with others in a fair manner  Competency to interact effectively with people and build effective intra- and intergroup relationships  Art of effective listening | | | | | | | | | | | | **Communication**  Constructive feedback to staff and patients;  Provide and receive constructive feedback  Abilities to receive and present constructive feedback  Ability to provide and receive constructive feedback for improvement  Effectively communicate with people based on their feelings, attitudes, motives, and attitudes.  Communication qualities  Communicate clearly and effectively with others  Skills to communicate effectively in dialogue to support employees    Engage confidently and constructively in verbal and non-verbal interactions with others  Establishment of effective interpersonal and inter professional communication  Determine the appropriate communication mode according to the situation    Assess the internal climate, including the values, feelings, and temperament prior to communication  Demonstrate listening skills and empathic understanding  Provide effective, actionable feedback  Ability to make your meaning clear to others  Transferring attitudes and beliefs clearly  Inform others; educate others  Effective communication  Effective verbal and non-verbal communication  Resolving communication barriers;  The art of oratory (having the power of speech and ability to present well | | | | | | | | | | **Negotiation**  Ability to negotiate effectively  Effectively use negotiation skills to mediate disputes and find appropriate and workable solutions  Negotiation capacity  Resolve conflicts among staff  Managing group conflicts within the organization  Facilitating conflict negotiation and dispute resolution  Manage conflict professionally and actively  Deal with difficult patients and their relatives  Skills in conflict resolution   Conflict management  Ability to identify and resolve organisational conflicts  Resolving conflicts and leading them toward organizational objectives  Ability to reach consensus when discussing issues and convince individuals  Managing conflicts of interest  Deal with difficult situations in my workplace  Managing conflicts  Managing workforce diversity  Resolving conflicts |
| 1. **Leadership**   Contains skills and abilities necessary to influence others, to motivate and mentoring staff. These competences are organisational internal, and involves direct influence such as communicating a shared vision and inspire team members to achieve it (Aitken & Von Treuer, 2014; Czabanowska et al., 2014; Li & Wivatvanit, 2016; Moghaddam et al., 2019; Ofei et al., 2020), motivating employees (Czabanowska et al., 2014; Dikic et al., 2020; Gunawan & Aungsuroch, 2017; Hargett et al., 2017; Kovačič & Rus, 2015; Moghaddam et al., 2019), encourage commitment and contribution (Gunawan, Aungsuroch, Fisher, & McDaniel, 2020; Gunawan, Aungsuroch, Fisher, McDaniel, et al., 2020b; Gunawan et al., 2019; Ireri et al., 2017; Lopes et al., 2020), mentoring, support and coaching (Dorji et al., 2019; Heinen et al., 2019), impact and influence (Aitken & Von Treuer, 2014; Garman et al., 2020; Gunawan, Aungsuroch, Fisher, & McDaniel, 2020; Hopkins et al., 2015; Kantanen et al., 2017; Moghaddam et al., 2019; Yakubu et al., 2019), present information convincingly (Kantanen et al., 2015), act as a role model (González García et al., 2020; Gunawan, Aungsuroch, Fisher, & McDaniel, 2020; Supamanee et al., 2011) and inspire staff (Gulati et al., 2019; Yakubu et al., 2019).  This category contains four sub-categories. (1) *Influence* is the most prominent sub-category of this category and illustrates how leadership differs from the categories management and soft HRM. It includes descriptions of direct influence as well as skills needed to motivate staff. For example: successfully influence subordinates, peers, and superiors in the realisation of organisational goals; influence people to work together as a team; encourage a high level of commitment to the purpose and values of the organisation; ability to promote motivation, professional interest, commitment and responsiveness; and ability to inspire every member to imbibe and work towards the organisational goals. The second sub-category, (2) *mentor*, is related to the first but includes descriptions that entail being a role model more than influencing and motivating staff directly. This sub-category entails descriptions like supporting and mentoring high potential staff; act as a resource person, preceptor, mentor/coach, and role model; give advice and coach health workers; be respected by the staff; model effective group process behaviour; and be a role model of professional and visioning behaviour. (3) *Leading* comprises of competence descriptions of leadership actions that involve the staff directly in their work. To delegate and assign tasks and responsibilities to staff based on their abilities is one example, others are: use leadership style and management techniques into activities; demonstrate servant leadership style; abilities to realise the potential development of the work community and employees of different ages; hold oneself and others accountable; and know when to consult clinic employees when making strategic decisions. The fourth sub-category, (4) *direction,* features skills and abilities to communicate strategy, vision, mission, and goal of the organisation to the staff, and to get the staff to work towards reaching those. Examples as follows: directing and channelling human behaviour toward the accomplishment of objectives; build organisational commitment and make staff feel goal-directed; be able to communicate specific programme targets to staff; understand policy and communicate a shared vision as well as inspire team members to achieve it. | | | | | | | | | | | | | | | | | | | | | | | | | |
| **Sub-categories of *leadership* with examples of descriptions:** | | | | | | | | | | | | | | | | | | | | | | | | | |
| **Influence**  Employee motivational techniques  Successfully influence subordinates, peers, and superiors in the realization of organisational goals  Impact   Influence  Motivating employees  Motivating others  Influence people to work together as a team to accomplish organisational goals  Inspire others on the team to achieve higher standards  Encourage a high level of commitment to the purpose and values of the organisation  Encourage high-level commitment  Influence decision-makers  Ability to inspire every member to imbibe and work towards the organisational goal  Motivation, and high level of commitment  Giving motivating feedbacks  Encouraging contribution  Provide encouragement, and the opportunity for people to engage in decision-making and to challenge constructively  Communication and influencing skills  Motivating subordinates  Ability to mobilise the capacities of others  Encourage others to feel ownership in the public health mission in the organisation  Encourage a high level of commitment to the purposes and values of the organisation  influencing and guiding others  Inspiration  ability to promote motivation, professional interest, commitment and responsiveness in oneself or others  Leading and motivating team members to visualise their highest level of performance  Creating interest in one’s own role raising awareness of and motivation in the role  Creating motivation, commitment, and responsiveness among employees to achieve determined goals | | | | | | **Mentor**  Mentor, advice, and coach  Model a healthy lifestyle to patients and subordinates  organisational commitment and confidence  Being role models  Support and mentor high-potential talent within the organisation  Acts as a resource person, preceptor, mentor/coach, and role model  Prepares through mentoring and coaching future generations of nurse leaders  Support and mentor high potential  Inspirational Leadership  Role modeling  Role models in terms of their demeanour and dress, their speech, taking responsibility, making sacrifices, and helping others  Be respected and liked  Respect from the nursing and healthcare team and being secure in life  representing positive attitudes and values  Model effective group process behaviours including listening, dialoguing, negotiating, rewarding, encouraging, and motivating  Coaching or mentoring staff in developing problem-solving skills  Becoming role model of professional and visioning behaviour  Acting as a mentor and role model for staff  Supporting and mentoring high potential talent  Manage as organisation leader with honesty, transparency and as a good role model | | | | | | | | **Leading**  Delegation of roles and responsibilities   Effectively  believe in the talent and potential of their employees  Assign tasks and delegate responsibilities to staff based on their abilities  Demonstrate a servant leadership style  Leading people and organisation:  Leading people and teams  Leading organisation  Leader quality  Use leadership style and apply management techniques into activities  Abilities to realise the potential development of the work community and employees of different ages  Demonstrate leadership qualities  Exhibit decision-making,  Hold self and others accountable  Communication skills  Respect, value and acknowledge the roles, contributions and expertise of others  promoting equality and diversity  Demonstrate managerial behaviors that are patient-centered and build concern for the organisation and staff  Know when to consult clinic employees when making strategic decisions  give credit to team members  Involve personnel in accomplishing the objectives  build consensus  Seek input of others | | | | | | | | | **Direction**  Clarify vision, mission and goal of hospital  Sharing the vision and mission  Develop and communicate vision  Providing direction  Communicating organisational goals  Directing and channelling human behaviour toward the accomplishment of objectives  Build organisational commitment and make nurses feel goal-directed  Able to communicate the specific programme targets to my staff  Implement my vision for health improvements in this clinic  Clarify vision, mission, and goal of SHPH precisely  Understand policy and communicate the policy to staff to implement it into practice  Present information convincingly  Create and communicate a shared vision for the future and inspire team members to achieve it  Managing team performance | | |
| 1. **Professionalism**   The category professionalism includes specific competences related to good and safe professional practices. It includes professional and clinical development (Aitken & Von Treuer, 2014; Dorji et al., 2019; Heinen et al., 2019; Munyewende et al., 2016), having clinical skills (Kantanen et al., 2015, 2017; Pihlainen et al., 2016; Supamanee et al., 2011; Walsh et al., 2019), establishing and organise communities of practice and interprofessional collaboration (Gunawan, Aungsuroch, Fisher, & McDaniel, 2020; Gunawan et al., 2019), be updated and inform on the resent trends and research within ones field (González García et al., 2020; Kovačič & Rus, 2015; Li & Wivatvanit, 2016; Murphy et al., 2016), hold and foster professional ethics and values (Ángel-Jiménez & Lopera-Arrubla, 2013; Czabanowska et al., 2014; González García et al., 2020; Gunawan & Aungsuroch, 2017; Heinen et al., 2019; Kakemam et al., 2020, 2021; Kitreerawutiwong et al., 2015; Lopes et al., 2020; Murphy et al., 2016; Walsh et al., 2019), continuous training and development of own and staff’s professional knowledge (Czabanowska et al., 2014; Gunawan, Aungsuroch, Fisher, McDaniel, et al., 2020a; Gunawan et al., 2019; Lopes et al., 2020; Moghaddam et al., 2019; Nazari et al., 2018), and strong commitment to patients needs and rights (Aitken & Von Treuer, 2014; Ángel-Jiménez & Lopera-Arrubla, 2013; Gunawan, Aungsuroch, Fisher, McDaniel, et al., 2020a; Gunawan et al., 2019; Hargett et al., 2017; Moghaddam et al., 2019).  Three sub-categories comprise this category. (1) *Professional practise and development* entails skills and abilities to work actively to develop, contribute to and enhance the professions, as well as own’s and staff’s professional knowledge, skills, and abilities. Examples of descriptions are enhancing the skills of staff; contribute to professional knowledge and evidence; participate in continuing education; acquire and stay current with the professional body of knowledge; ensure that the staff receive ongoing in-service training to perform their professional tasks; and keep professional colleagues informed about new developments in the professional field. The second sub-category, (2) *ethics and values,* is comprised of skills and abilities in ensuring ethical practise is maintained, that the right values are fostered and practised, as well as to sustain a commitment to the community. Examples of descriptions are ability to align personal and organisational conduct with ethical and professional standards; demonstrate understanding ethical rules and practices to protect the rights of customers; balance the values and priorities of both organisation and profession; maintain professional and ethical standards; responsibility to the patient and community; and commitment to help the deprived. Several competencies described skills, abilities, and practices to be patient centered, improve patient care and quality of services and were coded as (3) *patient care*. For example: go above and beyond expectations to focus on patients/patient care; commitment towards customers, valuing patients, ability to identify and meet their needs and respecting their rights; maintain sensitivity about patient issues and demonstrate proactivity before a problem arises; developing quality assurance and improving patient safety; and show genuine interest in patients. | | | | | | | | | | | | | | | | | | | | | | | | | |
| **Sub-categories of *professionalism* with examples of descriptions:** | | | | | | | | | | | | | | | | | | | | | | | | | |
| **Professional practice and development**  Commitment to lifelong learning and improvement  Enhancement of professional competences - Focus on enhancing the skills of employees  Professional development  Proficiency needed to work with instruments and procedures based on specific knowledge of a discipline  Specialist knowledge  Contribute to professional knowledge and evidence  Participate in continuing education and career planning  Acquire and stay current with the professional body of knowledge  Engages in advanced nursing practice and provide leadership for evidence‐based practice  Participates in professional organisations and activities that influence advanced practice nursing  Provides leadership in the development and integration of the nurse practitioner role within the healthcare system  Assumes responsibility for own professional development by pursuing education,  Learn the knowledge that can foster the development of nursing, such as research, ethics and law  Participating in professional committees and work groups  Contributing to a work environment where continual improvements in practice are pursued  Commitment to advancing the profession  Demonstrated professional roles  Entrepreneurial intention in the field of health  Previous experience in the field of health  Specific and technical knowledge and education in the field of health  Participating in continuing professional development and from experience and feedback  Clinical and technical know-how  Professional competence and credibility  Stay updated about new trends and developments in nursing  Stay updated about new knowledge and skills in nursing, as well as new solutions to complicated diseases  Demonstrate the spirit of devotion and passion to the nursing profession for reasons that go beyond money or status  Ensure that the risk of infection to patients in this clinic is minimised  Ensure that staff receive ongoing in-service training to perform their nursing tasks  Knowledge and skills of professional and clinical operations issues and professional credibility  Specialists’ requirements and current medical knowledge  Professional ethics and learning from mistakes and failures  Professional competence and credibility  Know how to practise nursing  Know how to lead practical nursing    Keep professional colleagues informed about new developments in the field of work  Definition of scope of professional practice standards  Competencies needed in practice, such as educating patients to care for and manage themselves at home, and in advanced nursing care to patients on the ward  Care for patients and family  Specific knowledge of nurse leaders about clinical leadership, management and nursing informatics  Continuous professional development opportunities  Possess effective professional development programmes and opportunities for staff  User care skills  Clinical skills  Standard nursing practice  Nurse research  Nursing theories  Care planning  Nursing training planning  Professionalism  Infection control practices  Managing the number and qualification of nurse staff based on the standard of hospital  Setting up learning  opportunities, trainings, continuing education and others related to nurse staff development  Explaining and demonstrating spiritual care practice to staff and patients  Referring effectively and articulately to other spiritual nursing care based on the situation and preference of staff and patient  Continuous training and development  Dedication to continuous learning  Providing opportunities for employees' professional development  Acquire and stay current with the professional body of knowledge  Ability to manage a ward using clinical skills  Achieving certification in an appropriate field/specialty  Managing self through continuing education and participating in nursing management research/training/course | | | | | | | | | **Ethics and values**  Ability to align personal and organizational conduct with ethical and professional standards  Demonstrate understanding ethical rules and practices to protect the rights of customers  Balance the values and priorities of both organisation and profession  Honesty  Have non-wavering values  Have a high level of moral  Demonstrate ethical and professional practice  Maintain professional and ethical standards  The desire to act in a way that is consistent with one’s values and beliefs  Know and apply medical ethical principles to difficult situations.  Professional and social responsibility  Ethical decision-making ability  Integrity and ethical conduct  The ability to align personal and organizational conduct with ethical and professional standards that include a responsibility to the patient and community  Know the ethics committee’s roles, structure, and functions  Adhere to ethical principles, professional roles, professional standards, and codes of ethical behaviour  Serve as an ethical guide for the organisation  Uphold and act upon ethics  Commitment to help the deprived  Social concerns  Identifying situations and giving safe and proper legal and ethical care  Managing legal and ethical issues is the ability of FLNMs to identify situations and give safe and proper legal and ethical care in a health care system  Acting with Professional Ethical Values  Maintain confidentiality of clients  Focus on the benefits of clients and the public  Respect the judgment of the working staff and other local organisations  Give advice on ethical practices and related laws on how to protect clients’ rights  Interact effectively with diverse individuals, groups and communities, demonstrate inclusive behaviours and practices  Demonstrate knowledge of cultural issues and practices, including cultural safety  Ethical commitment  Adhere to ethical legal and regulatory standards  Practice and promote professional accountability and social responsibility  Ethical principles  Giving safe and proper legal and ethical care  Reviewing and identifying the influence of cultural beliefs, values, and spiritual on nursing care.  Facilitating staff to provide spiritual nursing care to patient and families and respect their diversities and differences  Adherence to principles, values, beliefs, regulations  Perceiving principles, values, beliefs, and regulations  Considering ethical issues in decision making and organisational communication | | | | | | | | | | | **Patient care**    Focusing on the benefits and needs of patient and the public  Maintaining patient centeredness  Demonstrate managerial behaviours that are patient-centred (and build concern for the organisation and staff)  Maintain sensitivity about patient issues and demonstrate proactivity before a problem arises  Show genuine interest in my patients  Care for my patients’ well-being  The ability of clinical nurse leaders to connect all the needs and information of a patient for better nursing care  Consciously aware of providing patient care  Carry out comprehensive assessment of patients  Identifying and matching nurse staff competence with patient acuity  Commitment towards customers (valuing patients, ability to identify and meet their needs and respecting their rights  Commitment toward customers (Respecting patients' rights)  Go above and beyond expectations to focus on patients/patient care  Act upon critical situations of patients  Know the patient's perspective  Service orientation  Prioritising organisational interests on individual interests  Concern for quality  Strong commitment, and ability to advocate and negotiate, to achieve positive health and wellbeing outcomes for the client group  Implementation of best practice procurement  Best practice drug use and introduction of new drugs  Encourage my staff to display a caring attitude to patients according to the Batho Pele principles  responsibility to the patient and community  Quality and safety  Process improvement in terms of quality, increased focus on patients, patient safety and process management  Developing quality assurance and improving patient safety  Establish core values of the nursing team and develop the concept of teamwork | | | | | |
| 1. **Soft HRM**   Competence descriptions included in this category are related to arranging the work and well-being for employees (Aitken & Von Treuer, 2014; Heinen et al., 2019; Nazari et al., 2018; Pounder & Greaves, 2020); to create a productive and safe work environment and culture (Czabanowska et al., 2014; Dorji et al., 2019; Gulati et al., 2019; Heinen et al., 2019; Li & Wivatvanit, 2016; Lopes et al., 2020; Moghaddam et al., 2019; Munyewende et al., 2016; Nazari et al., 2018; Walsh et al., 2019). It contains skills and abilities to see and facilitate for the needs of the staff, developing and training their skills (Aitken & Von Treuer, 2014; Amini et al., 2018; Garman et al., 2020; Gunawan & Aungsuroch, 2017; Heinen et al., 2019; Hopkins et al., 2015; Kakemam et al., 2021; Li & Wivatvanit, 2016; Lopes et al., 2020; Munyewende et al., 2016; Pihlainen et al., 2016; Pounder & Greaves, 2020) , as well as provide formal education (Ángel-Jiménez & Lopera-Arrubla, 2013; Lopes et al., 2020), and to see everyone and their potential (Pounder & Greaves, 2020). This competence category is tied to the formal structures of the organisation and includes development discussions, postgraduate studies, professional guidance and development of professional skills (Fanelli et al., 2020).  This category contains four sub-categories. (1) *Staff development* includes to facilitate and take responsibility for the development, education and training of staff. Some examples from the descriptions are commitment to training for empowering new nurses; provide a professional career ladder to staff; focus on enhancing the skills of employees; and staff training and development. (2) *Facilitate* involves facilitating the professional practice and welfare of the staff. Examples includes determining and evaluating staffing needs; provide sufficient staffing and make flexible staffing plans according to the situation; facilitate self‐care management; the physical optimisation of the ward; and ensuring that staff are knowledgeable about what is expected from them at work. (3) *Work environment* includes taking responsibility and action to create a safe and thriving work environment. Examples of descriptions are monitor the work environment for potential safety issues that could affect staff and health services; encourage open communication and feedback as a means of coping with the nature of the working environment of health systems and to minimise the effect of dissenting voices on decisions and actions that need to be taken; and to create positive healthy (work)environments and maintain a climate in which team members feel heard and safe. Several competency descriptions involved personnel management, recruitment, and retention of personnel, as well as leadership style, and was coded (4) *managing human resources*. Examples are as follows: effectively manage team and individual performance, employ a supportive and collegiate management style; assign tasks and delegate responsibilities to staff based on their abilities; adapt leadership style to suit the situation; supportive supervision; and recruitment and retention techniques. | | | | | | | | | | | | | | | | | | | | | | | | | |
| **Sub-categories of *soft HRM* with examples of descriptions:** | | | | | | | | | | | | | | | | | | | | | | | | | |
| **Staff development**  Commitment to training for empowering new nurses  Planning and creating vision and mission for nursing, strategic planning and career planning for nurses, and planning nursing care  Developing skills of staffs,  Active participation in the empowerment of new nurses  Empowerment  Staff training and development  Talent development  Dedicated to the development of others  Provide mentorship, training and counseling to staff and encourage staff to achieve progress  Provide a professional career ladder to staff  Focus on enhancing the skills of employees  Participate in continuing education and career planning  Plans and implements training  Lead, develop and evaluate performance to build an effective team   Planning and Developing human resource  Give advice and coaching to health workers in detailed steps | | | | | **Facilitate**  Relieving staff spiritual distress  Determining and evaluating staffing needs  Ensure that staff receives ongoing in-service training to perform their tasks  Provide sufficient staffing and ensure patient safety and make flexible staffing plans according to the situation  Set the compensation and welfare for staff by performance appraisal  Go above and beyond to ease the workload of the team  Ensure that the staffs are knowledgeable in their roles, responsibilities, and job descriptions  Facilitate self‐care management  Smartly facilitating interpersonal and inter‑professional interactions  The smart engineering of the work process,  The physical optimisation of the ward  Paying attention to the spiritual needs of staff  Ensure that staff are knowledgeable about what is expected from them at work  Flexibility in nurses’ monthly work schedule | | | | | | | **Work environment**  Creating a whole systems environment in which employees can thrive  Encourage open communication and feedback as a means of coping with the nature of the working environment of health systems and to minimize the effect of dissenting voices on decisions and actions that need to be taken  Monitor the work environment for potential safety issues that could affect staff and health services  Creates a culture of ethical standards within organisations and communities  Create positive healthy (work)environments and maintain a climate in which team members feel heard and safe  Build organisational commitment and make nurses feel goal-directed  Creating an organisational climate  Ability for fostering environment of mutual trust  Creating an organisational climate that encourages teamwork  Creating a safe and spiritual work environment  Create a safe and healthy work environment  Flexibly house learning activities focused on the formation and clarification of professional and organisational values  Monitor the work environment for potential safety issues that could impact staff and patients  Promoting work environment | | | | | | | | | **Managing human resources**  Focus on the basic aspects of HRM  Implement corrective action in a timely manner in cases of noncompliance  Delegates and supervises tasks assigned to paraprofessional staff  Selecting and recruiting nurses  Recruitment and retention techniques  Managing People managing nursing personnel  Development and management of human resources and mastery of personnel  Facilitating staff retention  Management of colleagues based on competences  Supportive supervision  Demonstrate awareness of the human resources laws and regulations  Delegation was also an ethic practiced by officials who saw a nexus between this approach, team-work and enabling consultation  Adapt leadership style to suit the situation  Assign tasks and delegate responsibilities to staff based on their abilities  Have a system in place to manage staff absenteeism  The leader effectively manages team and individual performance, employing a supportive and collegiate management style | | | | |
| 1. **Management**   As opposed to both (3) *Leadership* and (5) *Soft HRM*, the sixth category excludes the interpersonal and relational aspects of leadership, and contains the steering part of the leader’s work, including directing and control. It employs principles of business, finance, economics, and health policy to develop and implement effective plans for practice-level and/or system-wide practice initiatives that will improve the quality-of-care delivery. This category includes skills and abilities in financing and budgeting such as cost management and financial analysis (Kakemam et al., 2021), plan, execute and evaluate projects (Liang et al., 2018), the ability to develop goals and plan tasks (Walsh 2019), setting priorities and establishing work plans (Dikic 2019), and to evaluate and manage employee efficiency and productivity (Lopes 2019) – amongst other.  The competence descriptions comprising this category are organised in five sub-categories. (1)  *Performance and evaluation* includes ability to result oriented, to manage and ensure performance as well as evaluating performance. Examples are identifying key criteria for performance evaluation; evaluating one's own activities based on standards for professional performance; evaluate accomplishments of expected results; outcome measurement and evaluation; and result orientation. (2) *Strategy* includes ability to develop and implement strategies, visions, and goals, and setting the direction. For example, establishing the organisation’s vision, mission, and goals; design and develop strategies to improve the quality of services; ability to implement approved programmes towards determined goals; make strategic decisions based on recognised values, priorities and resources; translate broad strategies into practical terms for others; and strategic thinking in personnel organisation. The third sub-category, (3) *resource management*, includes resource allocation, financial management, budgeting ability, and business literacy. Examples include actively participating in arranging a practical annual budget for the unit that includes volume, revenue, personnel, supplies, and capital equipment; mobilise processes to acquire resources; ability to use resources effectively and efficiently; estimating the department’s needs in terms of resources and facilities; and possess relevant experience in overseeing and/or managing financial structures and systems. (4) *Managing operations, organisation, and personnel* involves competencies to ensure an efficient and effective organisation and operations as well as staff utilisation, to improve processes, and control. This includes analysing the workflow of unit and identifying errors, causes, and its effects in nursing service; effectively assigning task responsibility and authority to subordinates; engaging in regular supervision or consulting with superordinate; ability to coordinate individuals and activities; foster integration and for expediting decisions and actions to counter bureaucratic hurdles and delays; and control and organise the clinical work for patient care at the level of self, team, and unit. The fifth sub-group, (5) *Administration* includes administrative tasks like establishing policy, systems, and structures, as well as knowing, apply and establishing rules and regulations. For example, getting involved in developing a policy brief and advocacy to policy makers; administer human, financial, material and information aspects of the health care business; actively participating in setting nursing practice standards or guidelines for nursing care in unit; monitor or audit the quality of processes; and apply relevant legislation and accountability frameworks specific to healthcare settings. | | | | | | | | | | | | | | | | | | | | | | | | | |
| **Sub-categories of *management* with examples of descriptions:** | | | | | | | | | | | | | | | | | | | | | | | | | |
| **Performance and evaluation**  Engaging in self-evaluation program in the hospital  Performance evaluation  Identifying key criteria for performance evaluation; Evaluating one's own activities based on standards for professional performance  Performance evaluation (ability to determine key criteria for evaluating actual performance on their basis)  Performance improvement  Evaluating nurses staff performance based on standard and key performance indicators  Result orientation  Evaluate accomplishments of expected results  Evaluate accomplishments  Evaluate the results obtained  Focused on objectives  The leader possesses a service improvement orientation  Deals effectively with performance management issues and challenges  Planning, evaluation and service improvement  Clarity of shared vision  Performance/outcome measurement and evaluation/Achievement orientation  supervise my staff through effective performance management  Use evaluation feedbacks to improve the quality of working staff  Monitor staff’s performance efficiency according to the KPI  Evaluation of organisation features leading to oscillation in staff needs  Organisation of remedial action | **Strategy**    Establishing organization's vision, mission, and goals  design and develop strategies to improve the quality of services, crisis management; risk assessments and analyses  Planning intelligence (ability to participate in planning and capability to understand action, tactical and strategic plans)  Strategic thinking (defining vision, goals and strategies)  Goal setting (ability to implement approved programmes towards determined goals)  Leading on vision and values  think through the ideas from an organisation and its vision for the future  creating visions,  formulating strategic plans  setting direction  being involved in developing policy briefs  Strategic vision  Make strategic decisions based on recognized values, priorities and resources  Strategic management  Translate broad strategies into practical terms for others.  Facilitate reassessment and adaptation of mission to match vision  Formulate, calculate, and analyze indicators  Elaborate strategic plan  understand and are committed to the underlying purpose for the consortium formation and service integration  focus on the mission of the organisation  providing directions  strategic planning  Design appropriate strategies to improve the quality of SHPH  Strategic orientation  Strategic thinking in personnel organization  demonstrate capability in assessment and analysis  Carry out assessment based on the SWOT matrix | | | | | | | | | **Resource management**  Resource allocation (ability to use resources effectively and efficiently)  Estimating the department’s needs in terms of resources and facilities  Financial management  Business literacy  Implementing cost benefit analysis approach in financial plan in the unit  arrangement and management of budget,  communicating financial plans and coordinating with supervisors.  Actively participating in arranging a practical annual budget for the unit that includes volume, revenue, personnel, supplies, and capital equipment  Marketing  Finance  Mobilize processes to acquire resources  Carry out resource audit activities  Carry out resource control  Determine required resources  Procure and mobilize the resources required  possesses relevant experience in overseeing and/or managing financial structures and systems (including managing funding cycles)  Financial skills  understanding operations and the importance of resource management  Provide cost-effective public health programs.  Use the mechanism of The SHPH administrative committee to gain financial support  Manage financial risks effectively  Organise access and use of assessment of cost-effectiveness | | | | | **Managing operations, organisation, personnel**    Engaging in regular supervision or consulting with superordinates  manage the workplace environments in organisation to efficient and safe work practices.  operational management abilities  Coordination (ability to coordinate individuals and activities)  foster integration and for expediting decisions and actions to counter bureaucratic hurdles and delays  Delegating roles and functions (emphasising meritocracy and considering staff's capabilities in distributing job duties)  Implementing corrective actions in case of malfunctioning  Execution  Problem-solving  Delegating roles and functions  Assigning work to senior nursing members  Planning and organisation  Coordinating with supervisor among departments  analysing workflow in the unit  Analysing the workflow of unit and identifying errors, causes, and its effects in nursing service  participating in setting nursing standards  Effectively assigning task responsibility and authority to subordinates  Delegate  Care management systems  Decision- making  Human resources management  Manage staff to effectively deal with change  Carry out programming of personnel under charge  Plan or calculate required personnel  Mobilize necessary personnel, processes, and resources  Apply the plan, do, verify, and act cycle  Monitor the execution of programs and projects  Design the program or project  Monitor compliance of the strategic plan  Execute actions that lead to accomplishing the plan  Mobilize necessary people and resources  is experienced at developing and implementing plans in accordance with priorities agreed by key stakeholders, and evaluates and updates plans regularly and systematically to ensure they meet current needs and priorities  possesses relevant experience in overseeing and/or managing workgroup structures and systems  possesses relevant experience in overseeing and/or managing  Possesses a sound understanding of, and experience with, people management frameworks systems and processes  Management of people.  Management of organisational systems and processes  Fostering organisational readiness  Organisational management  make effective evidence-based decisions  systems thinking  executing and delegating tasks  Able to predict and plan man power needs that respond to the health promotional plan of the SHPH  Planning of processes to be followed and settings  Organisation of effective practice  Organisation design to accommodate inevitable varying staff levels  Assessment of impact of temporary nursing personnel  Organisation greater staff self-sufficiency in staff varying needs  Implementation of processes to enhance stability in human resources  Fit of human resource skills to operational setting  activities that controlled and organized the clinical work for patient care at the level of self, team, and unit | | | | | | | | | **Administration**    Getting involved in developing a policy brief and advocacy to policy makers  Quality and safety orientation  administer human, financial, material and information aspects of the health care business  Establishing policy, systems and structures  Advocacy  Actively participating in setting nursing practice standards or guidelines for nursing care in unit  understanding and measuring care quality  Continually understanding and measuring quality of care in terms of structure, process, and outcome.  Legal aspects  Define and standardize nursing processes  Monitor or audit the quality of processes  possesses relevant experience in overseeing and/or managing clinical governance frameworks and practices  possesses relevant experience in overseeing and/or managing  communication systems;  information technology systems and procedures  possess effective organisational systems and processes associated with governance  Apply relevant legislation and accountability frameworks specific to healthcare settings  Apply appropriate techniques to solve community health problem  Provide guidelines, working procedure, projects and work activities that match the policy of SHPH  Demonstrate Efficiency: • preparation for meetings/presentations, etc.; and • focus on time management | |
| 1. **Organisational knowledge**   The competence category organisational knowledge contains competences that are organisational internal and involves knowledge of own organisation. For example, knowledge and understanding of organisation functions, decision-making systems and relationships (Walsh et al., 2019), knowledge of the institution’s activities, the institution’s standard operating procedures, rules and the institution’s services (Ofei et al., 2020), handle conflict of interest situations as defined by organizational by-laws, policies, and procedures (Lopes et al., 2020), and effectively navigate organisational structures, roles and relationships in order to achieve work goals (Liang et al., 2018).  This category has four sub-categories. (1) *Rules, regulations, and procedures* is a rather small sub-category when it comes to included descriptions but differentiates from for example the sub-category administration above by the pronounced knowledge of own organisation’s rules and regulations. Examples from the descriptions are knowledge of the institution’s standard operating procedures, rules, and the institution’s services; handle conflict of interest situations as defined by organisational by-laws, policies, and procedures; and navigate the politics of the organisation. The second sub-category, (2) *Organisation activities,* is about having knowledge of the internal life and activities inside the organisation. For example ability to think about different organisational issues systematically through recognising inter-relationships between them; have organisational awareness; identification of issues and risks; and to possess adequate knowledge of organisation issues. (3) *Organisational structure* is related to the previous one, and the two could be merged. In the competency descriptions of this sub-category, knowing how the organisation is structured and thereby how it functions is essential. Examples of descriptions are effectively navigate organisational structures, roles and relationships in order to achieve work goals; knowledge and understanding of organisational functions, relationships and decision-making systems; and creating an understanding between working departments. The fourth sub-category (4) *Operations* entails competency descriptions that address abilities to coordinate operations, knowledge of operations as well as develop plans for operations in the organisation. Examples are understanding roles and tasks of physician, nurses and allied health professionals; assessment of settings and scale of operation; knowledge of sequential and reciprocal task interdependencies; thoroughly knowing and understanding operations and available resources; and knowledge of the factors affecting the planning and organisation of human resources in the ward. | | | | | | | | | | | | | | | | | | | | | | | | | |
| **Sub-categories of *organisational knowledge* with examples of descriptions:** | | | | | | | | | | | | | | | | | | | | | | | | | |
| **Rules, regulations, procedures**    Knowledge of the institution’s standard operating procedures, rules and the institution’s services  Handle conflict of interest situations as defined by organisational by-laws, policies, and procedures  Production and implementation of clinical practice protocols  Understanding hospital's plans and policies  Navigate the politics of the organisation | | **Organisation activities**  Knowledge of the institution’s activities  Knowledge of organisation  Ability to think about different organisational issues systematically through recognising inter-relationships between them  Ability of an FLNM to plan for the organisation  Organisational awareness  Identification of issues and risks  Identification of institutional and non-institutional segments  Ability to identify and resolve organisational problems  Evaluating internal and external work environment  Identifying organisational conflicts  Possess adequate knowledge of organisation issues  Identifying and prioritising requirements | | | | | | | | | **Organisational structure**  Good organisational ability  Effectively navigate organisational structures, roles and relationships in order to achieve work goals  Knowledge and understanding of organisational functions, relationships and decision-making systems  Create understanding between working departments  Develop cooperation and coordination between nursing staff and hospital departments  Understanding of organisational structures | | | | | | | | **Operations**  Awareness of the nursing organisational chart  Understanding roles and tasks of physician, nurses and allied health professionals  Effective participation in various meetings held about the issue of nursing staff coordination  Assessment of settings and scale of operation    Assessment of impact of temporary nursing personnel  Knowing support services and workforce issues  Sequential and reciprocal task interdependencies  Coordination  Inter-sectoral coordination,  Intra-sectoral coordination  Thoroughly knowing and understanding operations and available resources, executive tasks  Managers organisational tasks and work content  Ability for the smart planning and organization of nurses  Knowledge of the factors affecting the planning and organization of human resources in the ward  Monthly planning based on the patients’ care requirements  The division of work based on nurses’ capabilities  Ability to provide intensive care with an insight  Full dominance over the ward and the patients’ treatment and care programs  Special insight  Demonstrate understanding of the highly professionalised health workforce | | | | | | |
| 1. **Technology**   This category includes skills and abilities to know of and be able to use information technology (Gunawan et al., 2019; Pounder & Greaves, 2020), but also to communicate literature and research to practitioners and professional networks (Kitreerawutiwong et al., 2015; Li & Wivatvanit, 2016), and in clinical practise to gain information about patients and utilise technology to support clinical practise (Gunawan, Aungsuroch, Fisher, McDaniel, et al., 2020a).  This category is divided into two sub-categories. (1) *Information technology* includes knowledge, skills and abilities to use and manage information technology. Examples are: use of information technology in patient care management and delivery, and practice; e-communication, stressing the significance of the use of information, communication and technology; Provide staff with access to databases to search research articles; effective use of latest communication technologies; demonstrate basic competency in statistics and Microsoft Office software; and evaluation and resolution of ethical and legal issues within healthcare systems relating to the use of information, information technology, communication networks, and patient care technology. (2) *Development* can be considered tentative as it does not contain many competency descriptions. But a few descriptions were centred around development and improving services by the use of technology, as well as utilising new technologies. Examples of these are appreciate the need for development and modernisation of the system of communication in order to keep up with rapidly evolving technologies necessary for a complex, dynamic system such as health; using latest technologies and their clinical applications¸ and effective use of latest communication technologies. | | | | | | | | | | | | | | | | | | | | | | | | | |
| **Sub-categories of *technology* with examples of descriptions:** | | | | | | | | | | | | | | | | | | | | | | | | | |
| **Information technology**  Information technology management  Use of information technology in patient care management and delivery and nursing practice,  E-communication, stressing the significance of the use of information, communication and technology  Communicate and share the latest nursing information with staff by information technology  Provide staff with access to databases to search research articles  Knowledge of information technology  Utilising technology to manage patients.  Integrating technology into nursing documentation  Use a computerised management system to record and analyse nursing data  Demonstrate basic competency in statistics and Microsoft Office software  Manage information system to achieve fast and convenient access  Advice for public health practitioner on data analysis  Propose and analyse problems with support of tools  Information systems and computers  Evaluation and resolution of ethical and legal issues within healthcare systems relating to the use of information, information technology, communication networks, and patient care technology. | | | | | | | | | | | | | 1. **Development**   Appreciated the need for development and modernisation of the system of communication in order to keep up with rapidly evolving technologies necessary for a complex, dynamic system such as health.  Evaluating the effect of Information Technology on patient care and delivery system  Using information system to support nursing practice  Using latest technologies and their clinical applications  Integration of technology in nursing documentation  Effective use of latest communication technologies | | | | | | | | | | | | |
| 1. **Knowledge of the healthcare environment**   Contains knowledge of the healthcare environment and system such as politics, other healthcare organisations, policy and the community, and their effect on own organisation. This includes to have knowledge and overview over political processes, goals, and directions, as well as knowledge of central stakeholders and their needs and expectations, the organisations position in a local and national context, and different groups’ function (Ireri et al., 2017; Kakemam et al., 2021; Li & Wivatvanit, 2016; Pihlainen et al., 2016). Further, knowledge and understanding of the laws, roles, and different functions of the political, social, and legislative systems (Pihlainen 2016).  Three sub-categories comprise this category. (1) *Healthcare environment impact* involves how policy, incidents, issues, community, or stakeholders have an impact on own organisation, or how the organisation adapts to issues in the environment. Examples of this are: envisaging potential impacts of decision making on operations, health care, human resources, and quality of care; implement plans and projects appropriately according to the nature of public health problems; development of a sound understanding and appreciation of the impact of the prevailing political culture at various levels of the system to facilitate effective and efficient decision making; analysing and understanding of demographic, political, social, technical, cultural and economic factors and their impact on the organisation; and understanding of the roles of key stakeholders. (2) *Interactions in healthcare* includes knowledge, skills and abilities regarding how organisations in the healthcare sector interacts and are interdependent, knowledge of the healthcare system, and how stakeholders interact. Examples as follows: demonstrate understanding of the roles of key stakeholders in health and how they interact; know the socioeconomic environment in which the organization functions; knowledge of interdependency, integration, and competition among health care sectors; know the health systems, structures, and the environment; and know the roles of nonclinical professionals in the health care system. (3) *Healthcare environment situation and development* entails knowledge and understanding of the situation in the healthcare sector, how it works, and identify development trends in healthcare as well as in the community that can affect the organisation. For example: knowledge of the health-care landscape due to the increasing change in the health-care environment; demonstrate knowledge of community development in the [client group] health context; implement plans and projects appropriately according to the nature of public health problems; possess adequate knowledge of healthcare environment and the organisation issues; know the implications of community standards of care and delivery of health care; and ensure that organisational practices are aligned with changes in the public health system and the larger social, political, and economic environment. | | | | | | | | | | | | | | | | | | | | | | | | | |
| **Subcategories of *knowledge of the healthcare environment* with examples of descriptions:** | | | | | | | | | | | | | | | | | | | | | | | | | |
| **Healthcare environment impact**  Envisaging potential impacts of decision making on operations, health care, human resources, and quality of care  Implement plans and projects appropriately according to the nature of public health problems  Identify the root causes of problems in the health services  the development of a sound understanding and appreciation of the impact of the prevailing political culture at various levels of the system was a special quality necessary to facilitate effective and efficient decision making  analysing and understanding of demographic, political, social, technical, cultural and economic factors and their impact on the organisation  understanding of the roles of key stakeholders    Health leaders demonstrate keen and quick insight in order to manage the affairs of the ministry and country at large, thus considering a whole systems approach  competency of envisioning as key to providing an analytic and strategic foundation for effective leadership practices from both the health system’s and national point of view  take into consideration public and political leadership in their country as this is very relevant and necessary for reflection in the communities and constituencies the healthcare services serve  Guides, initiates, and provides leadership in policy‐related activities to influence practice, health services and public policy  Articulates the value of nursing to key stakeholders and policy‐makers  knowledge and understanding of the laws, roles and different functions of the political, social and legislative systems  Being politically astute  External awareness  Global perspective or mindset  Knowledge of Australian health systems, structures and standards  able to navigate through complex and sensitive political issues (at the organisational, community, State and Federal levels  Foresee potential impacts and consequences of decision-making in both internal and external situations.  Evaluate and determine appropriate actions regarding critical political issues | | | | | | | | **Interactions in healthcare**  Knowledge of interdependency, integration, and competition among health care sectors  Provides leadership and acts as a liaison with other health agencies and professionals, and participates in assessing and evaluating healthcare services to optimize outcomes for patients/clients/communities  envisaging provided a better way of being and helped in an appreciation of the broader perspective of the interconnectivity of ministries of the government system and the need for sharing that vision among staff and stakeholders  the need to develop platforms for dialogue and negotiate, which pays dividends for the health system and the country at large  know the health systems, structures, and the environment  know levels of health care along the continuum of care.  know the roles of nonclinical professionals in the health care system  know the socioeconomic environment in which the organization functions  acts as a liaison with other health agencies and professionals  Demonstrates working knowledge of the healthcare system and its component parts, including sites of care, delivery models, payment models, and the roles of healthcare professionals, patients, caregivers, and unlicensed professionals  Demonstrate understanding of the roles of key stakeholders in health and how they interact  Identification of stakeholders  Identify and engage stakeholders in interdisciplinary projects to improve public health | | | | | | | | | **Healthcare/community situation & development**  Knowledge of the health-care landscape as being critical due to the increasing change in the health-care environment  Demonstrate knowledge of community development in the [client group] health context  Able to analyse factors or situations affecting public health problems  Implement plans and projects appropriately according to the nature of public health problems  Possess adequate knowledge of healthcare environment and the organisation issues  knowledge of healthcare environment  Knowing healthcare trends, the patient’s perspective (e.g. cultural differences, expectations).  Understanding the health system’s current business and operating frameworks as well as the dynamic context within which they operate (e.g., community, competitive, human resource, financial, legal, policy, and environmental  know the implications of community standards of care and delivery of health care  know regulatory and administrative environment  participates in assessing and evaluating healthcare services to optimize outcomes for patients/clients/communities  Identifies internal and external issues that may impact delivery of essential medical and public health services  Conduct performance assessment on the health workers on the basis of good governance  Apply information on health care and other related ones appropriately to identify public health problems  Mobilisation of appropriate specialists  Advocate and participate in public health policy initiatives at the local, national and/or international levels  Ensure that organizational practices are aligned with changes in the public health system and the larger social, political, and economic environment  Regular updating of programmes based on environmental change | | | | | | | | |
| 1. **Change and innovation**   This category involves competences where change and improvement are central. Pronounced skills in this category are to be able to identify and respond to different challenges and changes in the health services (Kakemam et al., 2021; Pihlainen et al., 2019; Walsh et al., 2019) to contribute to change processes, and also to adapt to change according to new politics and trends. It is also highlighted that healthcare leaders should arrange and lead change (Kakemam et al., 2020) and promote continuously learning and encourage innovative and diverse thinking in the organisation (Dorji et al., 2019).  Four sub-categories comprise this category. (1) *Leading change* includes competencies to lead and manage change and change processes successfully, especially to accommodate resistance, such as: managing employees' resistance toward change; involving key stakeholders in designing and implementing change; shared decision-making by involving staff in the process of change; identify the level of resistance to change and involve staff in building consensus on issues; assess readiness for change and plans accordingly; serve as a change agent and keep staff informed of change; and evaluate the processes and outcomes of change. Several competencies described skills and abilities to identify the need for change and be able to analyse environmental developments that will necessitate change. These were coded (2) *Need for change*. Examples include to have foresight, anticipate the need for resources to carry out initiatives and explore opportunities for the growth and development of the organisation; making effective changes to solve organisational challenges; actively contributing to change processes that lead to improving healthcare; identify opportunities for growth, innovation, change and development of the organization; and explore opportunities for growth. (3) *Improvement* includes being able to initiate change to improve the organisation and services. For example, to implement improvement activities gradually over time; willingness to challenge prevailing practice; analyse organisational systems for barriers and promote enhancements that affect client healthcare status; ability to make recommendations for service improvement; and keep up with the most up-to-date knowledge and information in order to improve service quality standards and service performance. The fourth sub-category, (4) *Change/innovation initiator and facilitator,* involves taking initiative and facilitate for change and innovation, such as to be enthusiastic local “change agents”; ability to use novel thinking in managerial planning; facilitating transformation; promote innovative thinking; encouraging diversity of thoughts; alertness to new opportunities; and encouraging improvement and innovation. | | | | | | | | | | | | | | | | | | | | | | | | | |
| **Subcategories of *change and innovation* with examples of descriptions:** | | | | | | | | | | | | | | | | | | | | | | | | | |
| **Leading change**  Managing employees' resistance toward change  Involving key stakeholders in designing and implementing change  Shared decision-making by involving staff in the process of change  Change management  Identify the level of resistance to change and involve staff in building consensus on issues  Serve as a change agent and keep staff informed of change  Act as a positive role model for innovation  Evaluate the processes and outcomes of change  Assess readiness for change and plans accordingly  Change leadership  Explain the need for change in an effective way  Ability for problem solving, forecasting, and planning strategies for overcoming obstacles  Ability for promoting and managing change  Change implementation and evaluation  Leader quality in change  Enabling and managing change | | | **Need for change**  Foresight, anticipate the need for resources to carry out initiatives and explore opportunities for the growth and development of the organisation  Making effective changes to solve organisational challenges  Actively contributing to change processes that lead to improving healthcare  Identify and communicate new system structures as needs are identified and opportunity arises  Explore opportunities for growth  Identify opportunities for growth, innovation, change and development of the organisation  Understand current public health issues and engage in systemic change to address them  Mentioning change as an opportunity  Ensure that organisational practices are aligned with changes in the public health system and the larger social, political, and economic environment  Service initiation and innovation  Provide visionary thinking on new initiatives in nursing  Demonstrate proactivity before a problem arises | | | | | | | | | | **Improvement**  Believing in implementing improvement activities gradually over time  Attitude towards continuous improvement  Focus on organisational change through strategic planning and systems thinking  Generate innovative solutions  Development-orientation; flexibility, responsiveness, willingness to challenge prevailing practice  Make recommendations for service improvement  Analyses organisational systems for barriers and promotes enhancements that affect client healthcare status  Demonstrate an ability to make recommendations for service improvement  Keep up with the most up-to-date knowledge and information in order to improve service quality standards and service performance | | | | | | | | | **Change/innovation initiator and facilitator**  Ability to use novel thinking in managerial planning  Ability to conduct effective activities towards facilitating appropriate modifications in an organisation  Respect diverse cultures and build upon the strength of diversity to bring about innovation and added value in the work environment  Serve as a driving force for change, including strategies of change  Facilitating transformation  Enthusiastic local “change agents”.  Skills to identify and respond to diverse challenges and changes  Promoting innovative thinking  Promote on-going learning  Encouraging diversity of thoughts  Innovation or creativity  Alertness to new opportunities  Innovate and create works to achieve performance  Question the status quo  Creating a climate of continuous service improvement  Encouraging improvement and innovation  Innovation thinking  Change preparation  Test ideas | | | |
| 1. **Knowledge transformation**   Describes skills and abilities necessary to acquire and utilising evidence and knowledge in practice and provide for knowledge sharing internal as well as external the organisation. This includes that the leader seeks information, and is able to learn outside of own context (Pounder & Greaves, 2020). Many of the included competence descriptions are also tied to decision making and problem solving based on evidence (Ireri et al., 2017; Kakemam et al., 2021; Kantanen et al., 2017; Liang et al., 2018; Pounder & Greaves, 2020; Walsh et al., 2019). It also includes evaluating information and its sources critically (Kakemam et al., 2021), gather sufficient information prior to solving a problem (Lopes et al., 2020) and use evidence to question and improve existing practice and processes (Liang et al., 2018).  This category includes three sub-categories. A few competency descriptions were interpretated as skills and abilities to facilitate for spreading knowledge to and among staff, especially to foster an open debate climate. These comprise the first sub-category (1) *Internal knowledge distribution.* Examples of descriptions included in the first sub-category are effectively share information and responsibility at different organizational levels; know how to promote the evidence-based decision-making process to staff; conceptualize and synthesize abstract ideas for staff and organization; and openness to real interchange with those involved in the organisation and encouraging open debate on all issues. Most of the competency descriptions in the category knowledge transformation were however coded to the second sub-category, (2) *acquire and utilise knowledge*. This sub-category involves skills and abilities to acquire knowledge from different sources, to utilise this knowledge in practice, to produce knowledge, as well as being able to critically assess sources. Examples are benefit from others’ experiences in the field of health and seek input of others; ability to collect and analyse valid information in an appropriate time; evaluate information and its sources; know how to produce new knowledge through scientific methods; keep up with the most up-to-date knowledge and information in order to improve service quality standards and service performance; and gather sufficient information prior to solving a problem. The third, (3) *evidence-informed practise* includes to utilise evidence; knowledge from multiple sources, own and other’s experience, in a broad spectrum of leadership and management duties. For example: implementation of best practice procurement; evidence based informed decision-making; gathering information to produce an evidence-based challenge to systems and processes in order to identify opportunities for service improvements; use timely and appropriate questioning/investigation to identify the nature of a problem, issue or opportunity; and integrate information from multiple sources to make decisions. | | | | | | | | | | | | | | | | | | | | | | | | | |
| **Subcategories of *knowledge transformation* with examples of descriptions:** | | | | | | | | | | | | | | | | | | | | | | | | | |
| **Internal knowledge distribution**  Openness to real interchange with those involved in the organisation and encouraging open debate on all issues  conceptualise and synthesise abstract ideas for staff and organisation  know how to promote the evidence-based decision-making process to staff  Synthesise and integrate divergent viewpoints  Effectively share information and responsibility at different organisational levels in pursuit of population-based goals | | | | **Acquire and utilise knowledge**  Competence in knowledge application activities: identifies current relevant scientific health information  Benefit from others’ experiences in the field of health  Seek input of others  Information literacy (ability to collect and analyse valid information in an appropriate time)  Evaluation of information and its sources  Collect data and information on health problems systematically to be analysed  Learn new systems, technologies, procedures  Apply information on health care and other related ones appropriately to identify public health problems.  Knowledge in legal and quality practices  Know how to produce new knowledge through scientific methods  Benefiting from the collective wisdom  Search information on public health or on epidemiology to be used in health and project planning  Anecdotal evidence in an environment where evidence-based decision making is not a prominent feature of the leadership and policy decision-making landscape  Ensuring accuracy and integrity of information  Evaluating information and its sources critically  Collecting and analysing data from internal and external sources relevant to each situation  Keep up with the most up-to-date knowledge and information in order to improve service quality standards and service performance  Asking and learning outside of the leader’s context  As part of the inquiry process, factual information is collated and expeditiously reviewed to determine a way forward  Seeking knowledge, facts and information through questioning  Gather sufficient information prior to solving a problem  Knowledge application activities  Identifies current relevant scientific health information, the translation of research in practice, the evaluation of practice, improvement of the reliability of healthcare practice and outcomes, and participation in collaborative research  Ability to integrate information  Gather sufficient information prior to solving a problem  Ability to distinguish information  Ability to judge issues based on facts and identify alternate processes  Collect and analyse data  Ability for transforming strategic plans into workable operational plans  Information seeking  Use of own and other people’s experiences  Having a strong knowledge base    Show concern about the trending topics of nursing research and incorporate research findings into practice | | | | | | | | | | | | | | **Evidence-informed practice**  Evaluation of practice against best practice  Implementation of best practice procurement  Evidence-based actions  Evidence-based decision making (ability to make decisions and find appropriate solutions based on valid evidence)  Integrate evidence-based medicine to guide my practice  Evidence based informed decision-making  Evidence appraisal  Promotion of evidence-based decision making  Evidence‐based practice  Evidence application and decision making  Analyse and evaluate information  Using evidence-based practice  Make effective evidence-based decisions  Gathering information to produce an evidence-based challenge to systems and processes in order to identify opportunities for service improvements  Decisions have to be made through inquiry  Proper inquiry needs understanding, knowledge, reasoning and critical thinking to keep decision making from being influenced by unsupported information  Use timely and appropriate questioning/investigation to identify the nature of a problem, issue or opportunity  Use evidence to question and improve existing practice and processes  Support and encourage colleagues and subordinates to use evidence to guide decision-making  Integrate information from multiple sources to make decisions  Demonstrating ability to integrate, analyse, and evaluate information from various sources to make decisions  Applying knowledge and evidence | | | | | | | |
| 1. **Boundary spanning**   While the category (9) *knowledge of health care environment* involves knowledge of how actors in the environment affect own organisation, *Boundary Spanning* includes to coordinate and cooperate beyond own organisation , share knowledge to the environment, and build and maintain networks in the environment (Garman et al., 2020; Liang et al., 2018), establishing information networks and ability to interact with peer organizations (Amini et al., 2018; Kovačič & Rus, 2015). Boundary spanning includes to get input from outside the organisation to inform decision making, to understand stakeholder needs, to maintain effective stakeholder relationships, to optimise the relationship between the leader’s control span and the departments, organisations, society and det the larger network the organisation is operating within (Czabanowska et al., 2014; Gunawan, Aungsuroch, Fisher, & McDaniel, 2020; Gunawan et al., 2019; Kakemam et al., 2021; Liang et al., 2018; Pounder & Greaves, 2020; Walsh et al., 2019).  Three sub-categories constitute this category. The first (1) *networking* overlaps to some degree to *interpersonal relations* as it includes creating and maintaining personal and organisational networks outside of the organisational borders. Examples of descriptions are: participate in relevant networks regional, national, and international; ability to understand stakeholder need and to maintain effective stakeholder relationships; know how to influence health care policy; take the initiative in establishing professional contacts with experts outside the organisation; and formal and informal networking. The second sub-category is related to the first, but involves to collaborate and build cooperative relationships with stakeholders or other organisations, to communicate and interact with external organisations. Examples of descriptions included in (2) *Interaction with environment* are: effective communication at different levels of the system as an important benefit and point of inquiry; ability to interact with peer organisations; collaborating with partners; build constructive collaboration between new divisions and across agencies; and optimising relationships between a leader’s span of control and the departments, organisations, communities and/or broader networks within which it operates. The third, (3) *communication to the environment* includes being able to communicate to the environment through media or other channels, and provide necessary information about the organisation to stakeholders, other organisations and the community. This includes: give interview or write articles to the media; ability to create international communication; inform community representatives about all the relevant clinic issues; participates in peer‐review activities e.g. publications, research, and practice; and communicate fiscal management expectations and outcomes to staff and other stakeholders. | | | | | | | | | | | | | | | | | | | | | | | | | |
| **Sub-categories of *boundary spanning* with examples of descriptions:** | | | | | | | | | | | | | | | | | | | | | | | | | |
| **Networking**  Participates in relevant networks; regional, national, and international  Organizational Awareness Network/Relationship  Formal and informal networking  Ability to understand stakeholder needs; to maintain effective stakeholder relationships  Invest time and effort in working and engaging with stakeholders  Build alliances, partnerships, and coalitions to improve the health of the community or population being served  Understand and apply effective techniques for working with boards and governance structures  Developing networks  Know how to influence health care policy  Take the initiative in establishing professional contacts with experts outside the organisation  Seek influential people  Network with others: focus on relationship-building  Networking and influencing  Possess strong pre-existing networks in the local health sector, the [client] sector and community.  Establish and maintain internal and external relationships appropriately  Provides leadership and acts as a liaison with other health agencies and professional  Build cross-agency relation  Ability to influence key decision makers who determine future government policies  Build academic relationships with nursing experts, and actively attend nursing programs  I take the initiative in establishing professional contacts with experts outside the organization  Establish and maintain a personal and professional support network | | | | | | | **Interaction with environment**    Optimising relationships between a leader’s span of control and the departments, organisations, communities and/or broader networks within which it operates  Carry out intra- and extra-institutional contacts  Involving key stakeholders in designing and implementing change  Community Collaboration  Effective communication at different levels of the system  Ability to interact with peer organisations  Collaborating with partners    Building constructive collaboration between new divisions and across agencies  Design and plan an accurate and reliable community health information management  Possess a well-developed ability to establish and maintain effective professional relationships to improve health and wellbeing outcomes for the [client group  Ability to negotiate with other institutions and organisations  Working in partnership with patients, careers, service users and their representatives, and colleagues within and across systems to deliver and improve services  Maintaining mutual understanding and trust with clients, communities, and other team members through effective communication  Persuade and cooperate with other organisations to support innovative health projects or activities in the community.  Search for support, resources and staff exchanges from other organisations and effectively employ them in health service work in the area.  Work effectively through intersectional collaboration with community hospitals, local government organisations, and community groups and parties  Encourage the process of building healthy public policies in community  Establish processes to promote a strong community with an awareness of health as a public concern | | | | | | | | | | | **Communication to the environment**  Interview or writing articles to media  Planning and implementing communication programs  Designing a long-term communication strategy  Ability to create international communication  Provides technical assistance and nursing consultation to health department staff, health providers, policy makers, and personnel in other community and governmental agencies and organisations  Effectively communicate the consortia’s shared vision, mission and values to staff, stakeholders and the community  Use the media to communicate routinely with target audiences regarding public health needs, objectives, accomplishments, and critical crises-related information  Inform community representatives about all the relevant clinic issues  Communicating fiscal management expectations and outcomes to staff and other stakeholders  Prepare and deliver business communications  Provide clear information, news and knowledge on healthcare to patients and the public  Possess effective communication skills to convince the public and the community to cooperate with the [clinic] in order to achieve the goals  Report the performance of the [clinic] to the public and private sectors and the community according to the KPI  Encourage the community to participate in solving health problems and monitor public health risk  Promote healthy lifestyle to all groups of people in the community  Share information between providers and network to improve healthcare  Write reports to meet the needs of different audiences  Produce written reports/materials, which are appropriate for both audience and purpose  Participates in peer‐review activities e.g. publications, research, and practice  Verbally and visually communicating and using factual data  Communicate news and information on health issues through community media or networks | | | | | | | |

**List of references:**

Aitken, K., & Von Treuer, K. (2014). Organisational and leadership competencies for successful service integration. *Leadership in Health Services*.

Amini, Z., Arasti, Z., & Bagheri, A. (2018). Identifying social entrepreneurship competencies of managers in social entrepreneurship organizations in healthcare sector. *Journal of Global Entrepreneurship Research*, *8*(1), Article 1. https://doi.org/10.1186/s40497-018-0102-x

Ángel-Jiménez, G., & Lopera-Arrubla, C. (2013). Relevance y level of application of management competencies in nursing. *Invest Educ Enferm [Internet] 2013 [Acesso Em 2017 out. 12]; 31 (1): 8-19*.

Czabanowska, K., Smith, T., Könings, K. D., Sumskas, L., Otok, R., Bjegovic-Mikanovic, V., & Brand, H. (2014). In search for a public health leadership competency framework to support leadership curriculum–a consensus study. *The European Journal of Public Health*, *24*(5), 850–856.

Dikic, M., Nikolic, D., Todorovic, J., Terzic-Supic, Z., Kostadinovic, M., Babic, U., Gacevic, M., & Santric-Milicevic, M. (2020). Alignment of Perceived Competencies and Perceived Job Tasks among Primary Care Managers. *Healthcare*, *8*(1), 9. https://doi.org/10.3390/healthcare8010009

Dorji, K., Tejativaddhana, P., Siripornpibul, T., Cruickshank, M., & Briggs, D. (2019). Leadership and management competencies required for Bhutanese primary health care managers in reforming the district health system. *Journal of Healthcare Leadership*, *11*, 13–21. https://doi.org/10.2147/JHL.S195751

Fanelli, S., Lanza, G., Enna, C., & Zangrandi, A. (2020). Managerial competences in public organisations: The healthcare professionals’ perspective. *BMC Health Services Research*, *20*(1), Article 1. https://doi.org/10.1186/s12913-020-05179-5

Garman, A. N., Standish, M. P., & Wainio, J. A. (2020). Bridging worldviews. *Health Care Management Review*, *45*(4), E45–E55. https://doi.org/10.1097/HMR.0000000000000243

González García, A., Pinto-Carral, A., Sanz Villorejo, J., & Marqués-Sánchez, P. (2020). Nurse manager core competencies: A proposal in the Spanish health system. *International Journal of Environmental Research and Public Health*, *17*(9), 3173.

Gulati, K., Madhukar, V., Verma, V., Singh, A. R., Gupta, S. K., & Sarkar, C. (2019). Medical leadership competencies: A comparative study of physicians in public and private sector hospitals in India. *The International Journal of Health Planning and Management*, *34*(1), e947–e963. https://doi.org/10.1002/hpm.2709

Gunawan, J., & Aungsuroch, Y. (2017). Managerial competence of first-line nurse managers: A concept analysis. *International Journal of Nursing Practice*, *23*(1), Article 1. https://doi.org/10.1111/ijn.12502

Gunawan, J., Aungsuroch, Y., Fisher, M. L., & McDaniel, A. M. (2019). Development and psychometric properties of managerial competence scale for first-line nurse managers in Indonesia. *SAGE Open Nursing*, *5*, 2377960819831468.

Gunawan, J., Aungsuroch, Y., Fisher, M. L., & McDaniel, A. M. (2020). Comparison of managerial competence of Indonesian first-line nurse managers: A two-generational analysis. *Journal of Research in Nursing*, *25*(1), 5–19.

Gunawan, J., Aungsuroch, Y., Fisher, M. L., McDaniel, A. M., & Marzilli, C. (2020a). Managerial Competence of First-Line Nurse Managers in Public Hospitals in Indonesia. *Journal of Multidisciplinary Healthcare*, *13*, 1017–1025. https://doi.org/10.2147/JMDH.S269150

Gunawan, J., Aungsuroch, Y., Fisher, M. L., McDaniel, A. M., & Marzilli, C. (2020b). Perceived managerial competence of first-line nurse managers: A comparative analysis among public hospitals. *Policy, Politics, & Nursing Practice*, *21*(3), 151–163.

Hargett, C. W., Doty, J. P., Hauck, J. N., Webb, A. M., Cook, S. H., Tsipis, N. E., Neumann, J. A., Andolsek, K. M., & Taylor, D. C. (2017). Developing a model for effective leadership in healthcare: A concept mapping approach. *Journal of Healthcare Leadership*, *9*, 69–78. https://doi.org/10.2147/JHL.S141664

Heinen, M., van Oostveen, C., Peters, J., Vermeulen, H., & Huis, A. (2019). An integrative review of leadership competencies and attributes in advanced nursing practice. *Journal of Advanced Nursing*, *75*(11), Article 11. https://doi.org/10.1111/jan.14092

Hopkins, M. M., O’Neil, D. A., & Stoller, J. K. (2015). Distinguishing competencies of effective physician leaders. *Journal of Management Development*.

Ireri, S. K., Walshe, K., Benson, L., & Mwanthi, M. (2017). A comparison of experiences, competencies and development needs of doctor managers in Kenya and the United Kingdom (UK). *The International Journal of Health Planning and Management*, *32*(4), Article 4. https://doi.org/10.1002/hpm.2357

Kakemam, E., Janati, A., Mohaghegh, B., Gholizadeh, M., & Liang, Z. (2021). Developing competent public hospital managers: A qualitative study from Iran. *International Journal of Workplace Health Management*, *14*(2), 149–163. https://doi.org/10.1108/IJWHM-07-2020-0120

Kakemam, E., Liang, Z., Janati, A., Arab-Zozani, M., Mohaghegh, B., & Gholizadeh, M. (2020). Leadership and Management Competencies for Hospital Managers: A Systematic Review and Best-Fit Framework Synthesis. *Journal of Healthcare Leadership*, *12*, 59–68. https://doi.org/10.2147/JHL.S265825

Kantanen, K., Kaunonen, M., Helminen, M., & Suominen, T. (2015). The development and pilot of an instrument for measuring nurse managers’ leadership and management competencies. *Journal of Research in Nursing*, *20*(8), Article 8. https://doi.org/10.1177/1744987115605870

Kantanen, K., Kaunonen, M., Helminen, M., & Suominen, T. (2017). Leadership and management competencies of head nurses and directors of nursing in Finnish social and health care. *Journal of Research in Nursing*, *22*(3), 228–244.

Kitreerawutiwong, K., Sriruecha, C., & Laohasiriwong, W. (2015). Development of the competency scale for primary care managers in Thailand: Scale development. *BMC Family Practice*, *16*(1), Article 1. https://doi.org/10.1186/s12875-015-0388-5

Kovačič, H., & Rus, A. (2015). Leadership Competences in Slovenian Health Care/Vodstvene Kompetence V Slovenskem Zdravstvu. *Slovenian Journal of Public Health*, *54*(1), 11–17.

Li, W., & Wivatvanit, S. (2016). A study of leadership competencies of first-line nurse managers in shanghai, china using Delphi technique. *Journal of Health Research*, *30*(5), Article 5.

Liang, Z., Howard, P. F., Leggat, S., & Bartram, T. (2018). Development and validation of health service management competencies. *Journal of Health Organization and Management*, *32*(2), 157–175. https://doi.org/10.1108/JHOM-06-2017-0120

Lopes, A. G., Narattharaksa, K., Siripornpibul, T., & Briggs, D. (2020). An assessment of management competencies for primary health care managers in Timor-Leste. *The International Journal of Health Planning and Management*, *35*(2), Article 2. https://doi.org/10.1002/hpm.2942

Moghaddam, N. M., Jame, S. Z. B., Rafiei, S., Sarem, A. A., Ghamchili, A., & Shafii, M. (2019). Managerial competencies of head nurses: A model and assessment tool. *British Journal of Nursing*, *28*(1), 30–37.

Munyewende, P. O., Levin, J., & Rispel, L. C. (2016). An evaluation of the competencies of primary health care clinic nursing managers in two South African provinces. *Global Health Action*, *9*(1), Article 1. https://doi.org/10.3402/gha.v9.32486

Murphy, K. R., McManigle, J. E., Wildman-Tobriner, B. M., Little Jones, A., Dekker, T. J., Little, B. A., Doty, J. P., & Taylor, D. C. (2016). Design, implementation, and demographic differences of HEAL: A self-report health care leadership instrument. *Journal of Healthcare Leadership*, *8*, 51–59. https://doi.org/10.2147/JHL.S114360

Nazari, R., Vanaki, Z., Kermanshahi, S. M., & Hajizadeh, E. (2018). The Meaning of Managerial Competency of ICU Head Nurses in Iran: A Phenomenological Study. *Iranian Journal of Nursing and Midwifery Research*, *23*(5), 363–370. https://doi.org/10.4103/ijnmr.IJNMR_132_17

Ofei, A. M. A., Paarima, Y., & Barnes, T. (2020). Exploring the management competencies of nurse managers in the Greater Accra Region, Ghana. *International Journal of Africa Nursing Sciences*, *13*, 100248. https://doi.org/10.1016/j.ijans.2020.100248

Pihlainen, V., Kivinen, T., & Lammintakanen, J. (2016). Management and leadership competence in hospitals: A systematic literature review. *Leadership in Health Services*.

Pihlainen, V., Kivinen, T., & Lammintakanen, J. (2019). Experts’ perceptions of management and leadership competence in Finnish hospitals in 2030. *Leadership in Health Services*.

Pounder, P., & Greaves, D. E. (2020). Impassioned leadership effectiveness: An assessment of leadership styles of top leaders in Caribbean healthcare systems. *International Journal of Public Leadership*.

Supamanee, T., Krairiksh, M., Singhakhumfu, L., & Turale, S. (2011). Preliminary clinical nursing leadership competency model: A qualitative study from Thailand. *Nursing & Health Sciences*, *13*(4), 433–439. https://doi.org/10.1111/j.1442-2018.2011.00649.x

Walsh, A. P., Harrington, D., & Hines, P. (2019). Are hospital managers ready for value-based healthcare? A review of the management competence literature. *International Journal of Organizational Analysis*, *28*(1), Article 1. https://doi.org/10.1108/IJOA-01-2019-1639

Yakubu, K., Dankyau, M., & Lodenyo, M. (2019). A Comparison of Leadership Competencies among Doctors Practicing in Public and Private Hospitals in Jos Metropolis of Plateau State, Nigeria. *Annals of African Medicine*, *18*(1), 23–29. https://doi.org/10.4103/aam.aam_9_18
